# Evaluating Ligand Modifications of the Titanocene and Auranofin Moieties for the Development of More Potent Anticancer Drugs

**DOI:** 10.3390/inorganics8020010

**Published:** 2020-01-26

**Authors:** Lauren Fernandez-Vega, Valeria A. Ruiz Silva, Tania M. Domínguez-González, Sebastián Claudio-Betancourt, Rafael E. Toro-Maldonado, Luisa C. Capre Maso, Karina Sanabria Ortiz, Jean A. Pérez-Verdejo, Janeishly Román González, Grecia T. Rosado-Fraticelli, Fabiola Pagán Meléndez, Fabiola M. Betancourt Santiago, Daniel A. Rivera-Rivera, Carlos Martínez Navarro, Andrea C. Bruno Chardón, Axel O. Vera, Arthur D. Tinoco

**Affiliations:** Department of Chemistry, University of Puerto Rico, Río Piedras Campus, Río Piedras, Puerto Rico 00931, USA;

**Keywords:** titanocene, auranofin, anticancer metallodrugs, ligand design, functionalized ligands

## Abstract

Over time platinum-based anticancer drugs have dominated the market, but their side effects significantly impact the quality of life of patients. Alternative treatments are being developed all over the world. The titanocene and auranofin families of compounds, discovered through an empirical search for other metal-based therapeutics, hold tremendous promise to improve the outcomes of cancer treatment. Herein we present a historical perspective of these compounds and review current efforts focused on the evolution of their ligands to improve their physiological solution stability, cancer selectivity, and antiproliferative performance, guided by a clear understanding of the coordination chemistry and aqueous speciation of the metal ions, of the cytotoxic mechanism of action of the compounds, and the external factors that limit their therapeutic potential. Newer members of these families of compounds and their combination in novel bimetallic complexes are the result of years of scientific research. We believe that this review can have a positive impact in the development and understanding of the metal-based drugs of gold, titanium, and beyond.

## 1. Introduction

**Figure 1.**
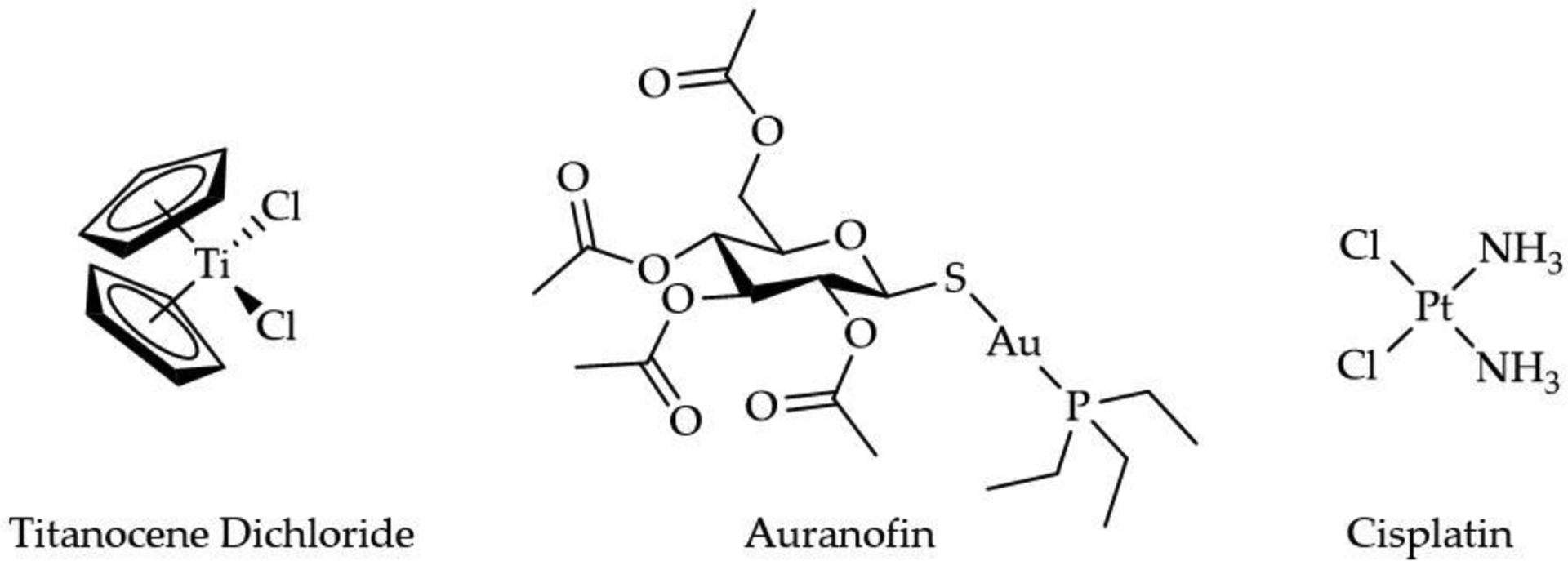
The chemical structures for titanocene dichloride, auranofin, and cisplatin.

Cancer is the second main cause of death throughout the world, claiming 9.56 million lives in 2017 [1]. In 2019, the American Cancer Society estimated that there would be 1,735,350 new cases of cancer and 606,880 deaths in the United States alone [2]. Chemotherapy, the use of chemical agents to treat cancer, remains one of the most effective anticancer strategies, with the platinum(II)-based compounds cisplatin, carboplatin, and oxaliplatin being widely used globally. Three others, nedaplatin, lobaplatin, and heptaplatin are used in specific countries but have not received FDA approval in the United States [3]. Cisplatin (Figure 1) was the first metal-based chemotherapeutic agent, which was discovered serendipitously by Barnett Rosenberg in 1965 [4]. Today, Pt(II) compounds are used to treat 40% to 80% of cancer patients [5,6]. However, as with other chemo drugs, their efficacy is hindered by clinical failures, including acquired resistance, a limited spectrum of activity, and high toxicity due to negative side effects [7]. A major source of the side effects associated with Pt-based drugs is the lack of exclusivity for cancer cells, resulting in unwanted biomolecular interactions with Pt(II). The formation of Pt(II)-DNA adducts in cancer cells is an intended target of the Pt(II) drugs as the adducts lead to suppressed DNA replication, but these adducts can also form in healthy tissue [8]. Even if the Pt(II) drugs could be selective for cancer cells, another issue is that the DNA reparation mechanism of the cells can attenuate the antiproliferative behavior of the metal. An overexpression of the multidrug resistance receptors (MDR) leads to a heightened efflux of the metal from the cell, significantly reducing the total metal content within cells [9].

Efforts are being made to improve the efficacy and cancer specificity of Pt compounds and to subsequently minimize their toxicity. In academia, the profound evolution of such compounds for anticancer treatment has been dictated by a better understanding of their antiproliferative and associated apoptotic mechanisms of action. It is now understood, for instance, that DNA is not the sole cellular target of Pt(II) (only about 1% of Pt(II) from cisplatin reaches DNA [10]), although it is certainly one of the more important ones [10]. Key to the knowledge development of the drug design of Pt compounds has been the recognition of the importance of the ligands to the activity of the compounds. Taking advantage of the coordination properties of the metal ion, cytotoxic ligands with a distinct mechanism of effect have been attached to the metal to increase the potency of the compounds [11,12]. Other ligands have been explored to develop anticancer *trans*-Pt(II) compounds [13] and even Pt(IV) compounds. Cellular reduction of the Pt(IV) ion to Pt(II) serves as an effective strategy to release cytotoxic agents bound to the metal center. Covalently conjugating certain drug delivery vectors onto the ligands has allowed increasing cellular uptake of Pt [14] and likely minimized the attenuation of the MDR system.

The discovery of the therapeutic properties of Pt revolutionized the drug field, reshaping the thinking behind drug design by demonstrating that a nonorganic species could impact human health and lead to a scientific renaissance to discover other metals for medical applications. Initially, the search was rather primitive and centered on empirically testing the therapeutic potential of different coordination compounds rather than a targeted approach. From this effort, titanium and gold, amongst other metals, emerged as very promising anticancer agents. In this review we will examine titanium(IV) and gold(I) in the anticancer field because their very different coordination chemistry yield distinct mechanisms of cellular effect, different even from the Pt(II) compounds. We will specifically focus on the titanocene (Cp_2_Ti^2+^) and auranofin (AF) families of compounds (Figure 1) to evaluate how understanding their mechanisms of effect, the aqueous chemistry and speciation of the metal ions, and the functional role of the ligands, have led to important evolution of the ligands, producing a new generation of anticancer agents with the potential to enter the drug market.

## 2. Titanocene Dichloride and its Ligand Evaluation

### 2.1 Biologically Relevant Ti(IV) Coordination Chemistry

The most stable and common oxidation state of titanium is +4; however, compounds in a range of lower oxidation states, −1, 0, +2, +3, are also known [15]. Nevertheless, these lower oxidation states are readily oxidized to Ti(IV) by air, water, or other reagents. In our aerobic atmosphere, particularly near neutral pH, Ti(IV) is favored, and most of the Ti in the environment is oxidized [15,16]. Even within the reducing environment of cells, Ti is expected to exist in the +4 state due to the absence of strong biological reducing agents [17]. The most prevalent coordination number of Ti(IV) is six, although four-, five-, seven-, and eight-coordinate compounds are known [15,18]. As a d^0^ metal ion, Ti(IV) coordination compounds are diamagnetic and do not have crystal field stabilization energy (CFSE). The lack of CFSE should make these compounds ligand exchange labile, but the high metal oxidation state decreases this lability [19].

**Figure 2.**
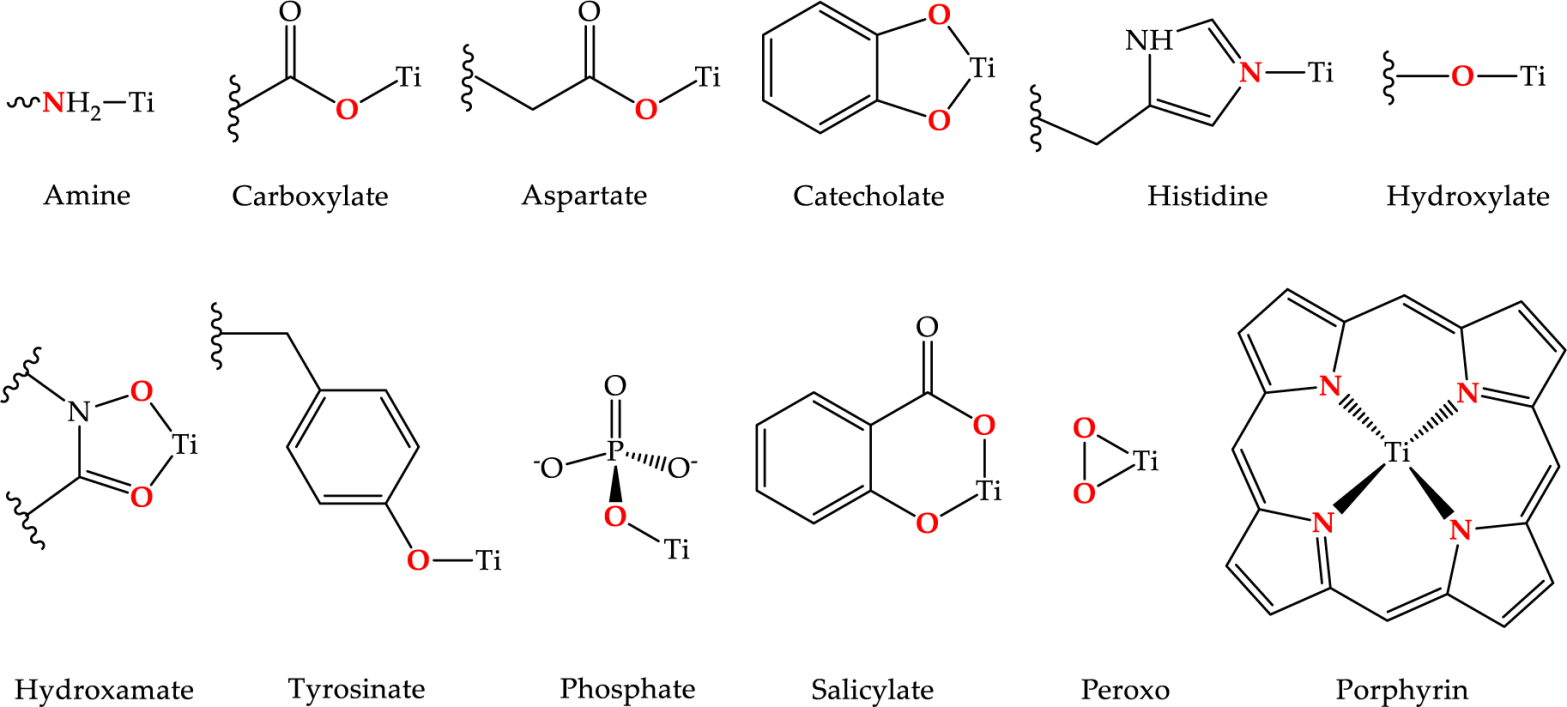
Typical Ti(IV) binding moieties of endogenous ligands with some representative coordination modalities. Reprinted from Coordination Chemistry Reviews, 363, M. Saxena, S.A. Loza-Rosas, K. Gaur, S. Sharma, S.C. Pérez Otero, A.D. Tinoco, Exploring titanium(IV) chemical proximity to iron(III) to elucidate a function for Ti(IV) in the human body, 109–125, Copyright (2018), with permission from Elsevier.

**Figure 3.**
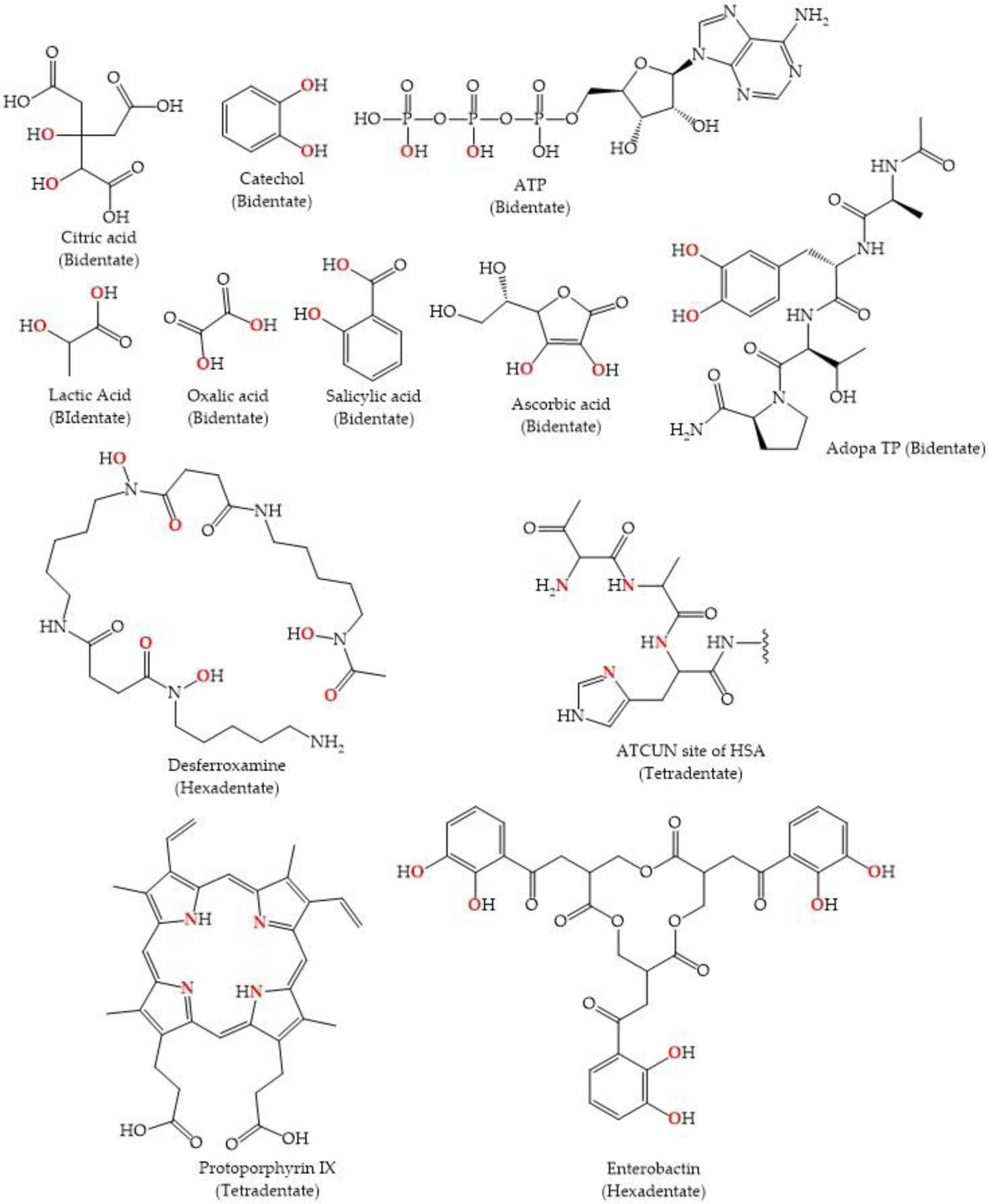
Some endogenous (or proposed) biological Ti(IV) chelators. Typical Ti(IV) binding moieties of endogenous ligands with some representative coordination modalities. Reprinted from Coordination Chemistry Reviews, 363, M. Saxena, S.A. Loza-Rosas, K. Gaur, S. Sharma, S.C. Pérez Otero, A.D. Tinoco, Exploring titanium(IV) chemical proximity to iron(III) to elucidate a function for Ti(IV) in the human body, 109–125, Copyright (2018), with permission from Elsevier.

The biological coordination chemistry of Ti(IV) is dictated by it being a very strong Brønsted-Lowry acid and a Hard Lewis Acid. The Brønsted-Lowry property is observed in the extensive hydrolysis that water molecules bound to Ti(IV) undergo [19]. Standard Ti(IV) salts are difficult to prepare from aqueous solutions, as they often yield hydrolyzed species, and at weakly acidic, neutral, or basic pH, will precipitate from the solution [18,20,21]. Due to its propensity for hydrolysis, well-characterized compounds of Ti(IV) that are prepared or stabilized in an aqueous solution are somewhat rare. There are examples of stable Ti(IV) complexes containing chelating ligands that exhibit partial hydrolysis in the form of the titanyl unit (TiO^2+^) [22,23] or oxo bridges [24]. As a Hard Lewis Acid, Ti(IV) is predominantly coordinated by biological chelators that contain oxygen-rich binding moieties, although binding by nitrogen-containing chelators has also been observed (Figures 2 and 3). The bioinorganic coordination chemistry of Ti(IV) largely mimics that of Fe(III) and is the subject of a recent review of ours [17].

### 2.2 History of Ti(IV) in Anticancer Research

Soon after the discovery of cisplatin, the search was on for other anticancer complexes with different central metals. The problem was that there was no established structure-activity relationships for metals other than Pt to help guide this mission [25]. The search was initially empirically driven, basically becoming the hunt for the next cisplatin-like compound. Ti(IV) was one of the first metals to be explored in the form of titanocene dichloride (Cp_2_TiCl_2_). In their initial work with the compound, Köpf and Köpf-Maier wrote, “We expected (Cp_2_TiCl_2_) to possibly show such an activity, since it contains a *cis*-dichlorometal moiety in the neutral complex, like the well-known antitumor agent (cisplatin)” [26]. Cp_2_TiCl_2_ very superficially resembles cisplatin. Its tetrahedral geometry (Figure 1) is unlike the square planar geometry of cisplatin. A second Ti(IV) compound, budotitane, belonging to the class of bis(β-diketonato) metal complexes, also caught the interest of researchers because of the cis arrangement of its ethoxide ligands. Fortunately, both Cp_2_TiCl_2_ and budotitane exhibited potent antiproliferative behavior against a variety of cell lines. We will focus exclusively on Cp_2_TiCl_2_ because it was the compound to advance the furthest from benchtop work to clinical testing and continues to be explored in modified forms today.

Cp_2_TiCl_2_ was initially tested against Ehrlich ascites tumor cells (murine mammary cancer) implanted in mice. The results were extremely promising because the compound had a cure rate in the therapeutic range of 80% [26]. Other studies demonstrated that it could be effective against Lewis lung carcinoma, colon B adenocarcinoma, B16 melanoma, and several other cancer types [27,28]. Cp_2_TiCl_2_ was also quite effective against cisplatin resistant cancer cell lines both in vitro and in vivo, which was an early clue that it operated via a different mechanism of action [29].

**Figure 4.**
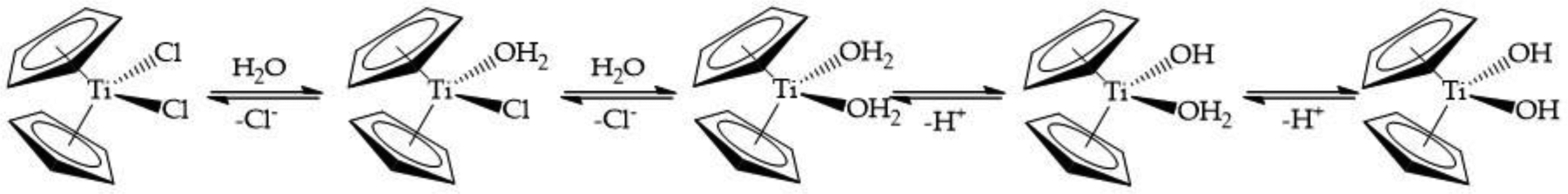
The hydrolysis of titanocene dichloride under acidic conditions focusing only on the dissociation of the chloro ligands.

The cytotoxicity of Cp_2_TiCl_2_ is predicated on the rapid hydrolysis of the compound in aqueous solution (Figure 4). The compound is poorly soluble in water, but once introduced into water with the help of a co-solvent such as DMSO, the chloro ligands are quick to dissociate. At pH 3.0, the first chloro ligand dissociates within seconds and the second chloro ligand dissociates with a half-life of fifty minutes [30]. These dissociation events are accompanied by aquation and hydrolysis. The cyclopentadienyl rings dissociate at a half-life of 57 h. Above pH 6.0, in the absence of any metal coordinating anion, Cp_2_TiCl_2_ undergoes virtually complete dissociation within seconds, yielding insoluble white precipitates that largely consists of titanium dioxide (TiO_2_) and polymeric hydrolyzed species containing substoichiometric equivalents of the Cp ligand [30]. It is believed that Cp_2_TiCl_2_ is a prodrug that once introduced into blood, requires facile delivery of Ti(IV) to the protein serum transferrin (sTf) for effective delivery into cells. STf is a bilobal glycoprotein present at ~30 μM in blood and is the primary transporter of iron(III) into all mammalian cells via endocytosis. Each of its lobes contains a metal binding site with identical coordinating amino acid moieties; Fe(III) is bound by two tyrosines, a histidine, an aspartate, and a carbonate anion that serves as a bidentate synergistic ligand. The protein is only 30% Fe(III) saturated [31] and thus can participate in the delivery of nonferric ions into cells [32–34].

**Figure 5.**
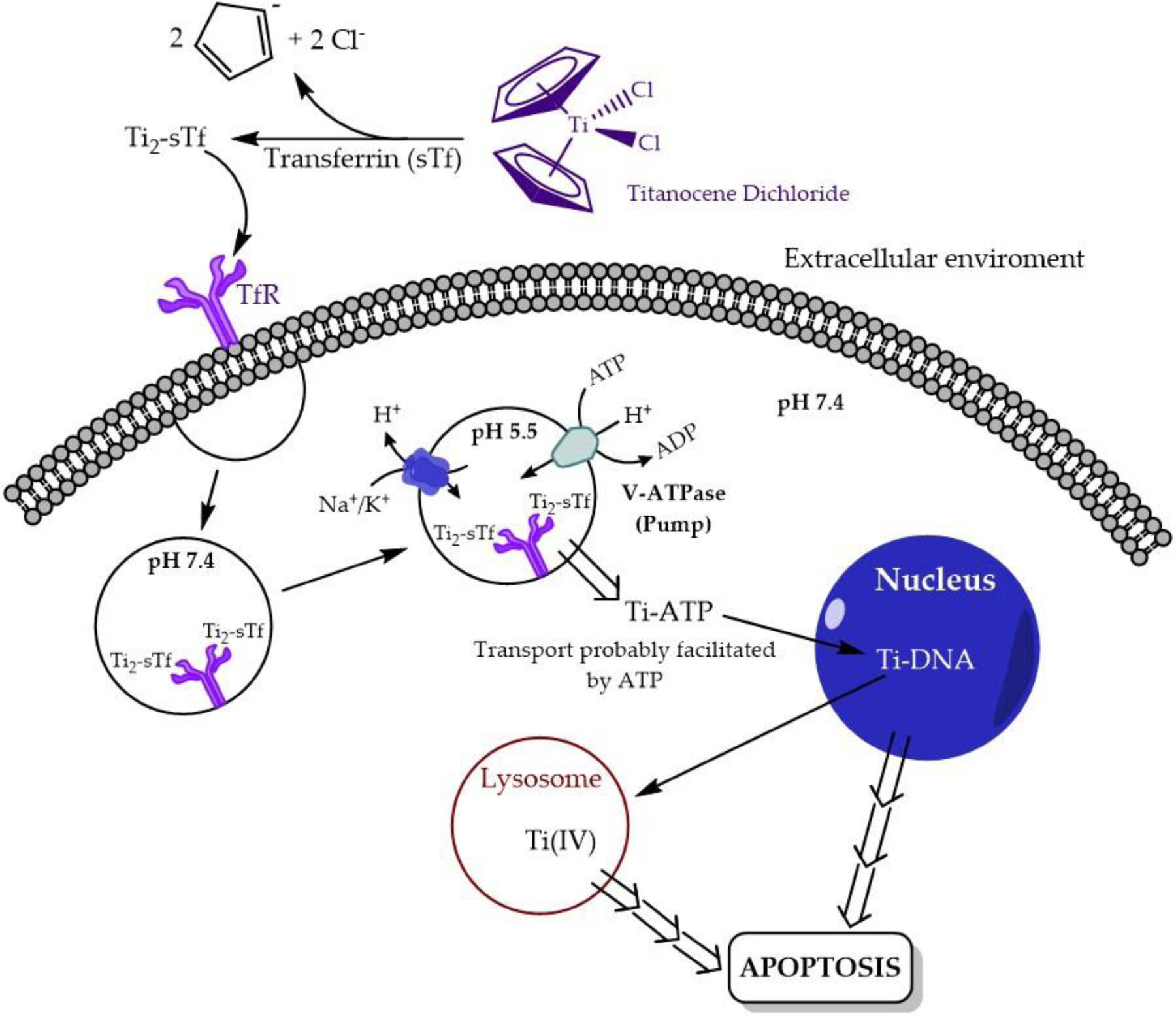
Proposed cytotoxic mechanism of action of Cp_2_TiCl_2_.

The discovery of Ti(IV) binding by STf [35–37] was very exciting because sTf is an excellent target for enhanced delivery of a drug to cancer cells versus normal cells. Due to their higher requirement for Fe(III), cancer cells overexpress the transferrin receptor, which is the protein responsible for the endocytotic uptake of metal-bound sTf into cells [38]. Although Ti(IV) can bind to sTf directly from Cp_2_TiCl_2_ as a hydrolyzed metal ion, it appears more likely to first be rapidly coordinated by the small anion citrate, which is present in blood at 100 μM, forming a Ti(IV) citrate complex (possibly Ti(citrate)_3_^8−^) that then delivers the metal ion to sTf (Figure 5) [39]. In this pathway, Ti(IV) is bound by sTf in a manner similar, but not identical to Fe(III). It is coordinated by the two tyrosines of the binding site and carbonate, and by one citrate molecule serving as an additional bidentate synergistic ligand. This interaction constitutes a ternary complex (Ti-citrate)_2_-sTf [39]. The citrate taking the place of the histidine and aspartate is likely a consequence of the Harder Lewis Acidity of Ti(IV). Presumably, in the absence of the citrate, an oxo or hydroxo moiety would take its place [17]. Once Ti(IV) is within the endosomes in cells, ATP is proposed to scavenge it from STf and transport it into the cytosol [37]. Ultimately, Ti(IV) accumulates in the nucleus and within cytosolic lysosomes (site of protein degradation). An electron-spectroscopic imaging (ESI) study of human stomach, colon, and lung adenocarcinoma cells, isolated from xenografted mice following Cp_2_TiCl_2_ treatment, demonstrated that within the nucleus and lysosomes, Ti(IV) colocalizes with phosphorus [40]. This colocalization is probably in the form of biomolecular phosphate coordination of the metal ion. Ti(IV) can bind to the phosphodiester backbone of DNA [41,42] and to phosphoproteins [43], and these interactions are key to its induction of apoptosis. Ti(IV) has the capacity to cleave phosphodiester bonds. Under acidic conditions, Ti(IV) can cleave ATP and produce inorganic phosphate [37]. However, it has been conclusively shown that Ti(IV) binding to DNA is incapable of cleaving the phosphodiester bonds and cause strand breaks [42]. Instead, the Ti(IV) binding causes detrimental structural changes that results in cell cycle arrest in the late S/early G2 phase and induces apoptosis at any phase of the cell cycle [44]. The apoptotic event results in the observed DNA breaks and a correlated increase in p53 levels, a protein that participates in DNA damage repair mechanisms [44]. Less clear is the potential interaction of Ti(IV) with phosphoproteins. Perhaps Ti(IV) binding to specific phosphoproteins could inhibit their functionality. Ti(IV) could also play a role in protein phosphorylation [17] and could thus significantly alter phosphorylation levels within cells [45]. Ti(IV) is also capable of blocking human topoisomerase interaction with DNA either by possible direct coordination to DNA or the topoisomerase [46], or by means of an indirect secondary effect [29]. This inhibition prevents the normal process of DNA replication. The exact biomolecular pathway leading to apoptosis remains elusive (Figure 5).

A less explored contribution to the mechanism of action of Cp_2_TiCl_2_ is the role that human serum albumin (HSA) may play. HSA is the most abundant serum protein (~600 μM). It possesses numerous binding sites capable of binding a variety of species, including metal ions, hydrophobic molecules, charged aromatic molecules, and drugs [47–49]. HSA binding of fatty acids causes protein conformational changes that enhances its binding of different species. The protein plays important functions in the pharmacodynamics of a drug in the human body, either increasing its lifespan in blood or sequestering it and helping to clear it from the system. It can also play a cellular transport function [47–49]. When Cp_2_TiCl_2_ was mixed at equimolar levels with HSA (1 mM), ^1^H NMR revealed that the Cp_2_Ti^2+^ moiety appears to bind to HSA (at an unidentified site) and remain stably bound for an indefinite amount of time. HSA, like sTf, provides a source of selectivity towards cancer cells because the accelerated metabolism of cancer cells causes them to catabolize albumin as a nutrient source much more than normal cells do and this results in a greater interaction with and possible uptake of albumin [50]. HSA binding of the Cp_2_Ti^2+^ moiety could thus provide a pathway for it to enter intact into cells. Alternatively, HSA could facilitate a direct contact of this lipophilic moiety with the cell membrane and enable entry via passive diffusion. It is uncertain, however, how long the moiety could remain intact within the aqueous environment of the cytosol (which is pH 7.4) and whether it could reach DNA intact. Nonetheless, the presence of the Cp ring could help to stabilize Ti(IV) coordination to DNA and more effectively damage it structurally.

### 2.3 Ti(IV) in Anticancer Clinical Trials

Budotitane was the first Ti(IV) compound to reach clinical trials. Two phase 1 trials were administered on cancer patients who were not responsive to other treatments. In the first trial, the dose limiting toxicity was nephrotoxicity (accompanied by nausea, vomiting, weakness, and malaise) [51], and in the second was cardiac arrhythmia [52]. In both studies, some patients experienced an impairment of their sense of taste. A tumor response to budotitane was not observed in either study, and the compound was not considered for further testing.

A total of five clinical trials were conducted to examine Cp_2_TiCl_2_. Three phase I trials involved patients with advanced solid tumors and evaluated the dose limiting toxicity of the compound. Christodoulou et al. identified 140 mg/m^2^ as the maximum tolerated dose (MTD) [53] whereas Korfel et al. recommended 315 mg/m^2^ [54] and Mross et al. recommended 240 mg/m^2^ [55]. The differences in recommendation had to do with the frequency of administration. The toxicities observed for Cp_2_TiCl_2_ treatment were comparable to those of budotitane. Additional reported toxicities included hepatic toxicity, blurred vision, hypertension, anorexia, and insomnia.

Cp_2_TiCl_2_ advanced to phase II testing. Lümmen et al. administered a dose of 270 mg/m^2^ every three weeks for six weeks to renal-cell carcinoma patients (*n* = 14), in which 78.6% of the patients received two cycles [56]. Patients showed signs of tumor progression and high toxicity while undergoing treatment. Kröger et al. also administered the same dosage to patients with metastatic breast cancer, but the compound failed to show significant antitumor activity against this population [57]. This study was halted due to several reasons (i.e., progression, toxicity), and the recommended dosage was lowered to 240 mg/m^2^ for future clinical trials. Despite promising in vitro data, none of the admitted patients in the phase II trials achieved a complete or partial response to Cp_2_TiCl_2_ therapy. Failure in the phase II trials resulted in the compound being removed from further testing.

### 2.4 Titanocene Dichloride Therapeutic Limitation due to Its Formulation and Speciation in the Body

The hydrolytic instability of Cp_2_TiCl_2_ can be a major deterrent to its therapeutic potential. The compound is simply too reactive and can readily transform under different solution conditions into different Ti(IV) species. For clinical trial testing, Cp_2_TiCl_2_ was prepared in was what termed a soluble, water stable formulation called MKT4, in which the compound was combined with the sugar alcohol mannitol. Buettner et al. propose that the mannitol can coordinate to the Cp_2_Ti^2+^ moiety in a doubly deprotonated fashion forming a neutral Cp_2_Ti(mannitol) compound that is far more stable than Cp_2_TiCl_2_ [58]. The MKT4 formulated Cp_2_TiCl_2_ exhibits cytotoxic behavior against several cancer cell lines about on par with the parent compound [58]. Solutions of Cp_2_TiCl_2_ prepared in methanol or ethanol and left to equilibrate after some time will transform into Ti(IV) species with bound Cp and alcohol ligands such as [Cp_2_Ti(OMe)]^+^ and [CpTi(H_2_O)(OH)]^+^ as detected by electrospray ionization-mass spectrometry (ESI-MS) [59]. These species formed by aging in the alcohols exhibit far stronger cytotoxic potency against HCT116 (human adenocarcinoma) cells than freshly prepared Cp_2_TiCl_2_. They also result in elevated metal uptake in the cells. Ravera et al. suggest that these relatively more stable species may permit the Ti to exist in a lipophilic cell permeable form that could result in higher accumulation within cells [59].

The problem still remains that titanocenyl mannitol or alcohol byproducts are quasi-stable. In the environment of blood, they are likely to be biotransformed (transiently) by citrate into a Ti(IV) citrate complex and then ultimately converted into the ternary complex (Ti-citrate)_2_-sTf [39]. This ternary complex does not display cytotoxic behavior at low μM concentration [23]. Citrate and sTf have been shown to be able to work synergistically to attenuate the cytotoxicity of anticancer Ti(IV) complexes by inducing their dissociation [39]. This is not to say that Ti(IV)-bound STf is incapable of being cytotoxic. The issue is a matter of concentration. At a very high concentration, and nearing mM amounts, the complex displays antiproliferative behavior [60], implying that a certain concentration threshold needs to be met in order for cellular Ti(IV) levels to reach a high enough level to detrimentally impact cells. Even if the Cp_2_Ti^2+^ could bind to SA, the Cp_2_Ti–SA 1:1 complex also requires very high concentration (near mM) for antiproliferative behavior [23,60], which could account for why Cp_2_TiCl_2_ exhibits high μM IC50 values against numerous cell lines [44]. Cell media is typically supplemented with 10% fetal bovine serum, which contains citrate and biologically active serum proteins capable of interacting with the titanocene moiety. In their seminal work to unlock the mechanism of action of Cp_2_TiCl_2_, Christodoulou et al. did not detect any apoptotic behavior until examining the compound at mid to high μM concentrations [44].

### 2.5. Titanocenyl Modification and Encapsulation to Alter Method of Transport for Improved Cytotoxic Potency

Extensive studies have been performed to substitute the chloro groups and modify the substituents of the cyclopentadienyl rings of Cp_2_TiCl_2_ to improve solubility, aqueous stability, and cytotoxicity of the family of titanocene compounds. These efforts have achieved varying degrees of success, and this catalog of work has been the subject of excellent reviews by Harding et al., Caruso et al., Hamilton et al., De la Cueva-Alique et al., and Santiago et al. [28,29,61–64]. Rather than revisiting these assessments, we will instead focus on select studies in which efforts were made to modify the ligand platform to prepare titanocenyl derivatives that may exploit alternative transport mechanistic routes for cellular uptake.

**Figure 6.**
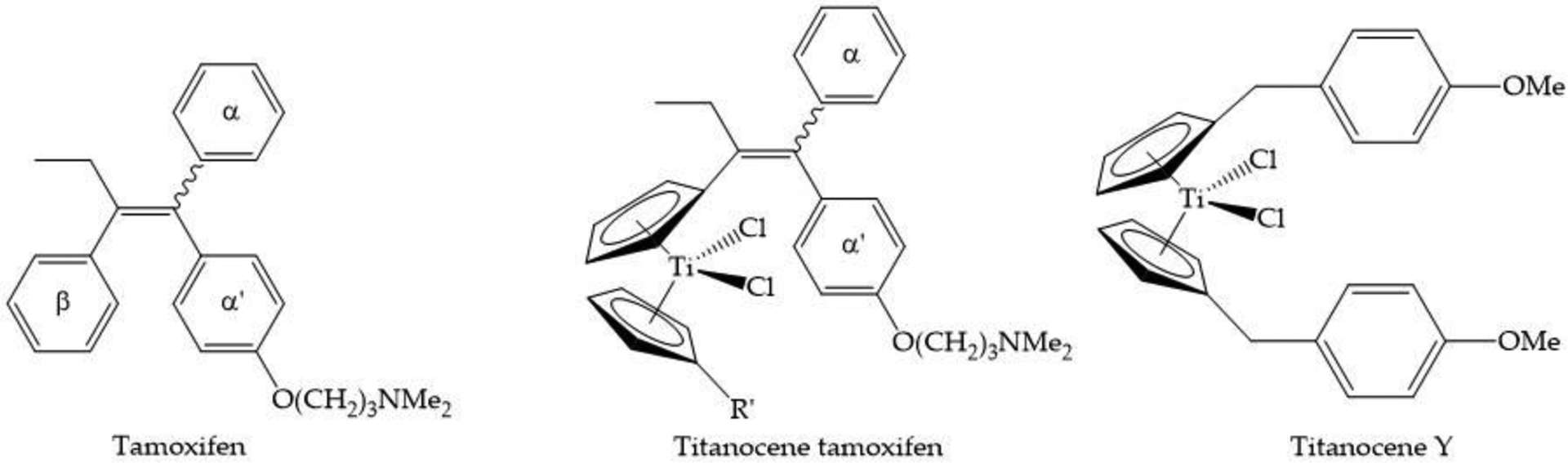
The chemical structures for tamoxifen, titanocene tamoxifen, and Titanocene Y.

Top et al. sought to vectorize titanocene by appending a tamoxifen-like moiety as an alkylated substituent of one of the Cp rings (Figure 6) [65]. Tamoxifen is a selective estrogen receptor modulator (SERM) and the objective was to examine whether a tamoxifen-like modification of Cp_2_TiCl_2_ would enhance its specificity and activity against the hormone dependent breast cancer cell MCF7. Surprisingly, the tamoxifen derivative produced a proliferative effect, as did the parent Cp_2_TiCl_2_ compound itself, both within nM to low μM concentration range. It was concluded that one of the hydrolysis products of Cp_2_Ti^2+^ including fully Cp dissociated Ti(IV) must exert an estrogenic effect and that the incorporation of a tamoxifen-like modification of one of the Cp rings was not enough to overcome this hydrolytic behavior. It is important to note that at high concentrations, Cp_2_TiCl_2_ is antiproliferative against MCF7 (IC_50_ = 570 μM). Interestingly, the titanocene derivative titanocene Y (Figure 6) featuring alkylated derivatives of aromatic groups on both Cp rings, in the vein of the type of modification achieved with tamoxifen, does not exhibit a proliferative effect on MCF7 cells [66]. Titanocene Y is a far more hydrolytically stable complex, which could account for this difference in behavior.

**Figure 7.**
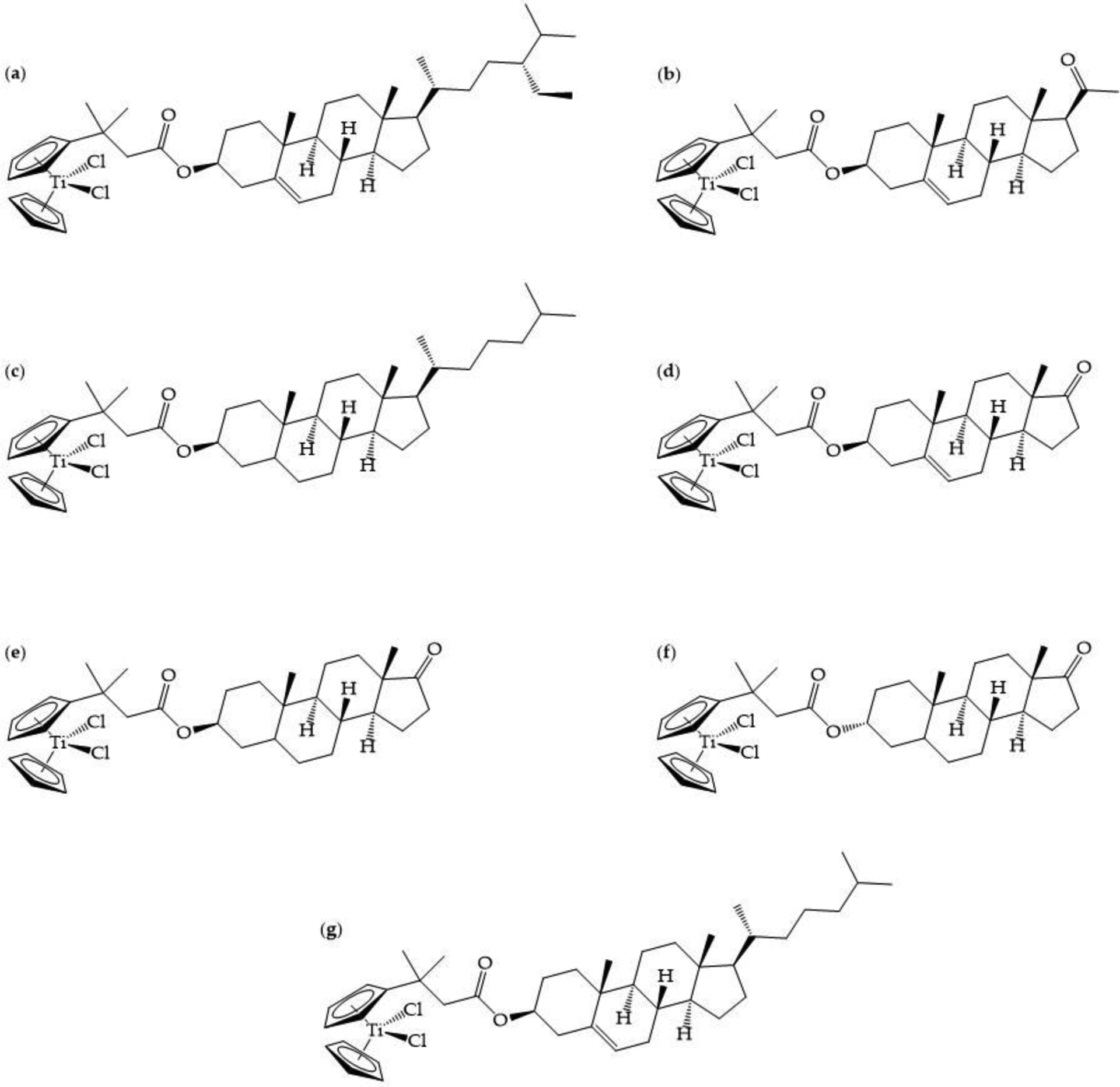
The chemical structures for titanocenyl clionasterol (**a**), pregnenolone (**b**), dihydrocholesterol (**c**), dehydroepiandrosterone (**d**), epiandrosterone (**e**), androsterone (**f**), and cholesterol (**g**) [67].

In a strategy similar to Top et al. [65], Gao et al. synthesized steroid-functionalized titanocenes, with modification of one of the two Cp rings, and determined their activity against MCF7 and colon cancer HT29 cells following 72 h of incubation [67]. The calculated IC_50_ values for the titanocenyl compounds of clionasterol, pregnenolone, dihydrocholesterol, dehydroepiandrosterone, epiandrosterone, androsterone, and cholesterol (Figure 7) ranged from 16.2–200 μM against HT29 cells and 13–200 μM against MCF7 cells. These values are much lower than the IC50 values determined for Cp_2_TiCl_2_ against HT29 (413 μM) and MCF7 (570 μM). No proliferative behavior, however, was observed at the low μM concentration range like Top et al. observed in tamoxifen-like modification of Cp_2_TiCl_2_ [65]. Cholesterol-functionalized titanocenes (clionasterol, dihydrocholesterol, and cholesterol derivatives) exhibited much lower activities (IC_50_ > 200 μM) than the activities of sex steroid-functionalized titanocenes (IC_50_ < 50 μM). This significant and selective increase in activity suggests that the steroid functionalities are facilitating cellular uptake. Gao et al. demonstrated with computational methods that steroid-functionalized titanocenes could bind the estrogen receptor alpha (ERα; PDB 1A52) [68]. They showed that the steroids on their own docked the protein analogously to the steroid hormone β-estradiol, surrounded by the amino acid residues Glu-353, Arg-394, His-524, Leu-387, and Met-388. However, the steroids conjugated to the titanocenes docked the protein via hydrophobic interactions in a fashion different from β-estradiol. While this result was a very promising finding, Gao et al. report that the steroid-functionalized titanocenes partially decompose in water after a few hours. Nonetheless, even after dissociation, the steroids appear to be able to interact with the titanocenyl moiety and still enable ERα binding of this group [67]. This study reveals a route for titanocenyl uptake through a plasma membrane estrogen receptor.

**Figure 8.**
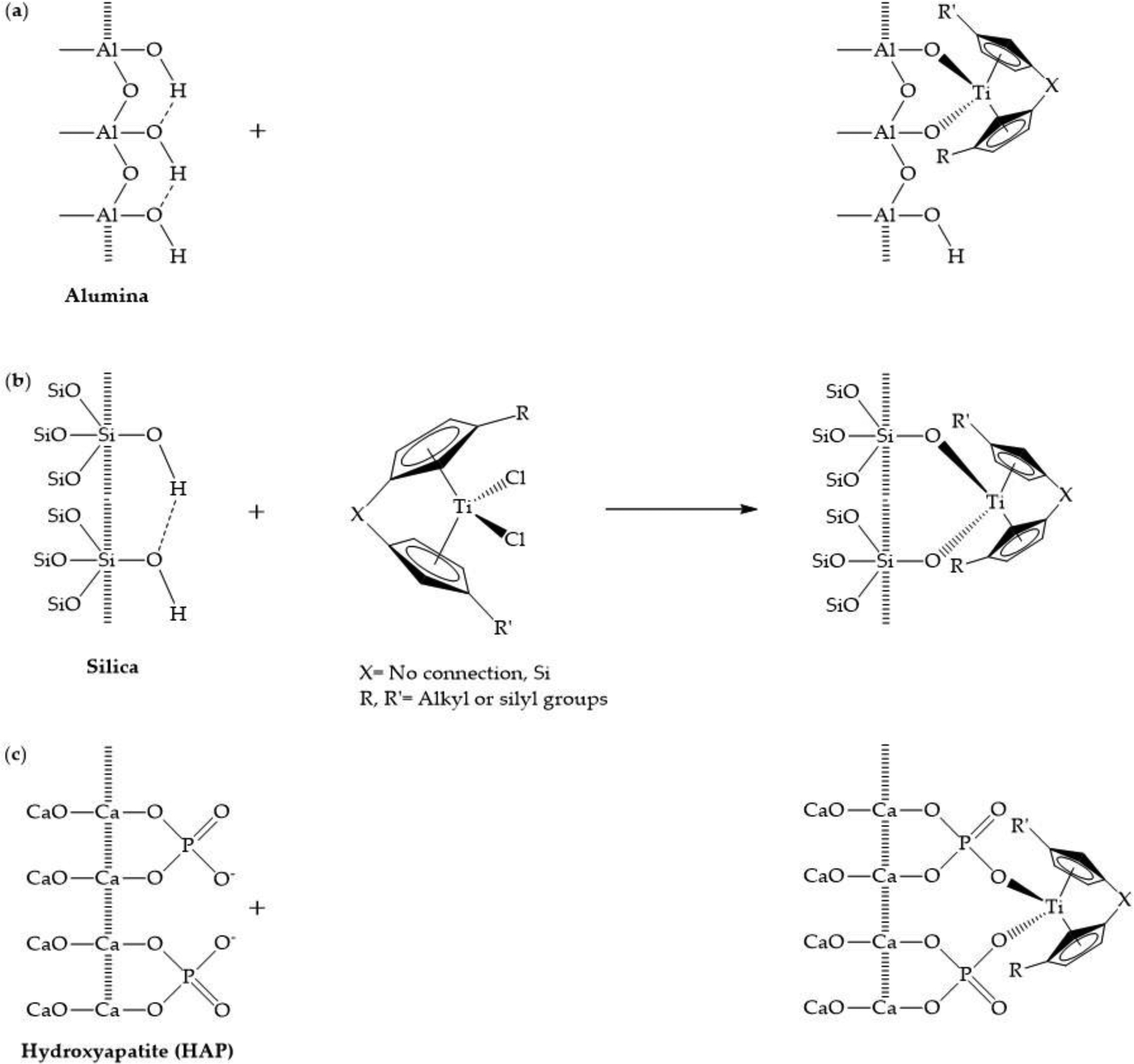
The titanocenyl moiety coordinating onto alumina (**a**), mesoporous silica (**b**), and dehydroxylated hydroxyapatite (HAP) (**c**).

In a series of studies, a different approach was taken to functionalize the titanocene moiety to not only stabilize the compound and enhance its cellular uptake but to also apply it to specific cancers [69–72]. Titanocene and select derivatives were grafted onto the surface of ceramic materials, including mesoporous silicas, alumina, and hydroxyapatite, that can be used as components of synthetic hard tissues, such as bone fillers or prosthetics (Figure 8). The objective is to be able to apply these materials for the treatment of bone tumors and for any remaining surface-bound metallodrugs to prevent reappearance of these tumors. This extensive work has been recently reviewed [63,73] but we wish to highlight a few key findings. The cytotoxicity of the ceramic materials and their titanocenyl grafted counterparts are measured in terms of M_50_ values, the quantity of material in μg/mL necessary to reduce the cell population’s growth by 50%. The ceramic materials alone exhibit very weak antiproliferative behavior, whereas the titanocene grafted material are moderately to strongly antiproliferative [69–72]. Ceballos-Torres et al. conducted a study of titanocenes grafted onto the mesoporous silica-based material KIT-6 [74]. In this work they acknowledged that the relative proportion of titanocene is not identical in all nanostructured materials. They calculated the cytotoxic activity of all of the functionalized materials in terms of Ti M_50_ index, the quantity of anchored Ti needed to inhibit normal cell growth by 50% [74]. Using this approach, they were able to determine a set of these functionalized materials that were highly selective toward cancer cells and displayed potent cytotoxicity. They then characterized their mechanism of cytotoxicity. It was observed that these materials are more strongly apoptotic than their “free” titanocenyl counterparts. The most promising ones are able to stimulate Bax-α proapoptotic expression and decrease Bcl-cl anti-apoptotic expression, and moderately inhibit the activity of PARP-1, a protein that participates in DNA damage repair, through cleavage [74]. They also observed that as in previous studies [70,71], the titanocenyl groups do not dissociate from the materials to interact with DNA. Instead there appears to be a direct interaction between the functionalized materials and DNA through electrostatic interactions. Also of significance, titanocenyl functionalized materials are able to increase the cellular uptake of Ti from about 0.4 to 4.6% of total available Ti (from free titanocenyl moiety) to 4 to 23% [74].

The addition of a vector on the titanocenyl framework may not be a necessity to facilitate enhanced cytotoxicity as can be observed with titanocene Y (Figure 6), one of the most active second generation non-vectorized titanocenes. A number of studies have been conducted to evaluate its mechanism of action against different cell lines. Cuffe et al. found that it can cause double-strand breaks and induce apoptosis in the human prostate cancer cell lines PwR-1E, 22Rv1, DU145, and PC3 [75] although likely not via Ti(IV) induced cleavage of the phosphodiester bond of the DNA backbone [42]. They treated PwR-1E and PC-3 cells with titanocene Y for 6, 12, 24, and 48 h and noted no significant effect on the ratio of G_1_, S, or G_2_ phases of the cell cycle [42]. Bannon et al. examined the effect of titanocene Y on A431 cells (epidermoid carcinoma) and saw a different behavior in terms of effecting the cell cycle [76]. Titanocene Y caused an accumulation of cells in the G_2_/M phase and a decrease of cells in the G_1_ phase at 24 h before the appearance of a pre-G_1_ peak at 48 h. They also found that titanocene Y (50 μM) induces caspase-3 and −7 activation of apoptosis in these cells. Treatment of A431 xenograft mice with titanocene Y (40 mg∙kg^−1^day^−1^) for five consecutive days caused a 40% inhibition in mean tumor volume [76].

**Figure 9.**
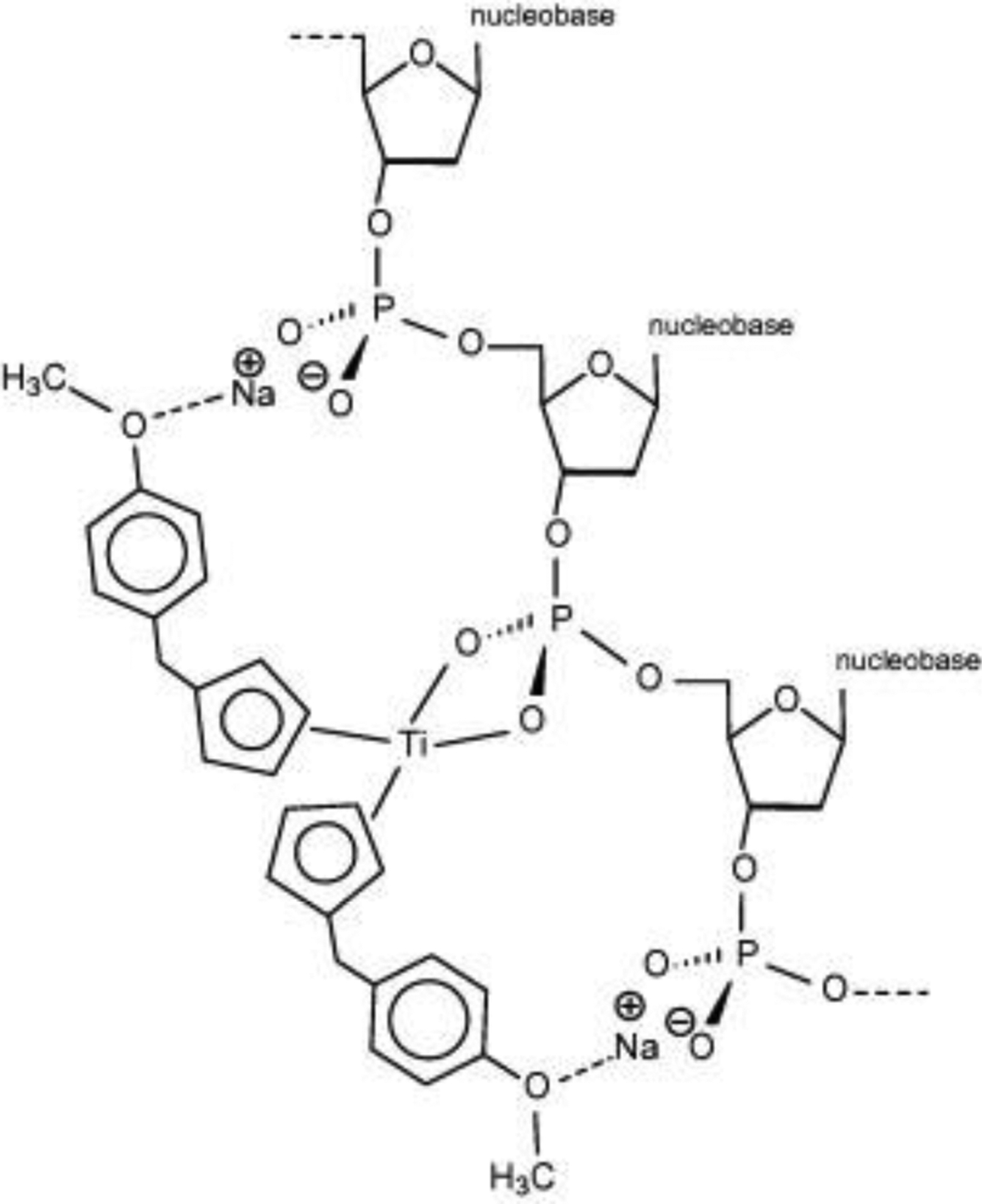
The calculated structure for the coordination of the (η^5^–C_5_H_4_–CH_2_–C_6_H_4_–OCH_3_)_2_Ti^2+^ moiety to the phosphobackbone of DNA. Reprinted from Journal of Inorganic Biochemistry, 104, A. Erxleben, J. Claffey, M. Tacke, Binding and hydrolysis studies of antitumoural titanocene dichloride and Titanocene Y with phosphate diesters, 109–125, Copyright (2018), with permission from Elsevier.

Although the identity of the active form of titanocene Y is not yet known, Tacke et al. proposed that the titanocenyl moiety may be able to bind directly to the DNA phosphate backbone. A calculated structure of the DNA-Titanocene Y adduct shows Ti(IV) coordinated bidentate to a phospho group. The titanocenyl moiety is stabilized via a Na^+^ salt bridge interaction between the methoxy groups of the aromatic pendants of the Cp rings and two phospho groups on adjacent sides of the phospho group binding the Ti(IV) (Figure 9) [42]. This potentially strong interaction could facilitate greater structural damage to DNA than merely free Ti(IV) binding or even Cp_2_Ti^2+^ binding by impeding topoisomerase interaction and thus inhibiting DNA replication [46]. HSA binding of the titanocenyl moiety of titanocene Y may be imperative to its cytotoxicity in some cells. Titanocene Y demonstrates antiproliferative activity against MCF7 cells at low μM only in the presence of albumin [66]. Schur et al. studied the effect of serum proteins on titanocene Y transport into cells [77], which elucidates insight into the active form of the compound. Titanocene Y is much more antiproliferative against MCF7 (IC_50_ = 4.1 μM) and HT29 (IC_50_ = 5.9 μM) cancer cells than titanocene (IC_50_ > 500 μM in both cases). These cell viability experiments were performed using serum containing cell media. Using high-resolution continuum source atomic absorption spectrometry (HR-CS AAS), they determined that titanocene Y binds DNA (290 pmol Ti/μg DNA) and albumin (76% bound) although to a lower extent than titanocene (320 pmol Ti/μg DNA, 98% bound) [77]. When cell culture media contained serum, intracellular titanium in cells incubated with titanocene Y (10 μM) increased 20 times after 27 h but remained below 1 nmol Ti/mg cellular protein. Cells incubated with Cp_2_TiCl_2_ (10 μM) in the presence of serum exhibited virtually no detectable titanium uptake. Cells incubated with titanocene Y (10 μM) without serum exhibited a significant increase in intracellular titanium after 27 h (4 nmol Ti/mg protein). This finding supports what we have already established that Ti(IV) from Cp_2_TiCl_2_ and titanocene Y binds strongly to serum transport proteins. For titanocene, sTf and HSA binding of either free Ti(IV) or the titanocenyl moiety, respectively, is likely to impact its cytotoxic potential. It is not clear why no Ti(IV) uptake could be detected except if uncontrolled hydrolysis of the compound occurred in this study resulting in a speciation that affects the nature of how these proteins interacted with the metal ion. For titanocene Y, lower Ti cellular uptake in the presence of serum may not necessarily impede its cytotoxic potential. Titanocene Y is highly stable in water with a half-life of more than a week but its speciation at pH 7.4 has not been reported. In the absence of serum, it may transform into hydrolyzed species that can be taken up by cells but may not result in Ti(IV) in the form of the titanocenyl moiety binding to DNA. In the presence of serum, the titanocenyl group is likely stabilized by rapid and strong binding to albumin. The cellular delivery of this group from albumin appears to be kinetically slow but perhaps a larger % of the titanocenyl moiety is able to bind at DNA, resulting in a more effective cytotoxic behavior. This would explain why titanocene Y shows antiproliferative behavior against MCF7 cells only in the presence of albumin [66].

Studies that have probed manipulating the titanocenyl ligand platform to deviate from the sTf model of Ti(IV) cellular uptake have led to development of more potent anticancer titanocene compounds. As demonstrated with the small molecule titanocene Y, simple modifications may me enough to make a more powerful and selective drug and is currently the most promising compound to advance Ti(IV), as a monometallic species, back to clinical trials.

## 3. Auranofin and its Ligand Evaluation

### 3.1. Biologically Relevant Au(I) Coordination Chemistry

Under biological conditions, gold (Au) can be found in an oxidation state of +1 and +3. In the reducing environment of cells, Au would predominantly exist in the +1 state. For the purposes of this review, we will focus on pertinent Au(I) coordination chemistry. Au(I) has a completely filled outer electronic shell ([Xe]4f^14^d^10^), is diamagnetic, and, like Ti(IV), has zero CFSE. Also like Ti(IV), the structure of Au(I) complexes are determined by steric effects of the ligands and simple electrostatic interactions that occur between ligands in the coordination environment of the metal ion. However, because Au(I) is a large cation with a low oxidation state, its outer electronic distribution is easily polarized, making it a Soft Lewis Acid, which prefers binding to Soft Lewis Bases. Due to electrostatic reasons, Au(I) has a higher stability in a linear two-coordinate geometry when its ligands are anionic. Au(I) can also form three-coordinate trigonal complexes and four-coordinate tetrahedral complexes. The higher coordination number is achieved with neutral ligands. Au(I) complexes with monodentate ligands in solution are extremely labile. This lability can be fine-tuned with the use of chelators [78].

### 3.2. Brief History of the Medical Use of Gold

Evidence dating back to 3000 BC suggests that the Egyptians used gold in purifying tonics while the Chinese, as early as 2500 BC, used it in medicines and tinctures for healing [79]. The formal use of Au as a treatment for medical conditions may be credited to two professors at the University of Montpellier in the 19th century: Andre-Jean Chrestien and Pierre Figuier [80]. Together, they worked on the formulation and advocated for the use of Au compounds, such as Au sodium chromide for the treatment of tuberculosis (TB) [80]. In 1890, German bacteriologist Robert Koch found that Au cyanide K[Au(CN)2] stopped tubercle bacillus from reproducing [81]. Due to this discovery, Au compounds were then evaluated as a treatment for TB. Many TB patients developed rheumatoid arthritis (RA), and it appeared that the Au cyanide alleviated some of the symptoms. However, it was found that the compound was not an effective therapeutic for TB, but was indeed for RA.

In 1960, The Empire Rheumatism Council, now known as Versus Arthritis, confirmed Au salts were an effective treatment for RA. This treatment became known as chrysotherapy. In 1972, Sutton, McGusty, Walz, and DiMartino published a study characterizing a series of Au compounds with trialkylphosphine ligands [82]. These complexes exhibited antiarthritic activity after oral administration to adjuvant arthritic rats. The triethylphosphine Au complexes were found to be the most effective, of which auranofin (AF) was particularly active [82]. AF is a linear Au(I) compound, in which the Au(I) is coordinated to the aforementioned trialkylphosphines and an S-glycosyl group (2,3,4,6-tetra-*O*-acetyl-1-thio-β-D-glucopyranose) (Figure 1).

Finklestein et al. were the first to perform clinical studies with AF (labeled SK&FD-39162) in 1976 [83], evaluating eight patients over six months. AF was administered in the form of 3 mg capsules either twice or three times a day for the first three months and then were given placebo for the remaining three months. Clinical improvement was observed starting at the fifth week of treatment. At the beginning of the trial, there were a total of 60 swollen joints and by week 15, there were only 9 [83]. In this study, no negative effects were observed but the sample size was too small to draw any definite conclusions. Other clinical trials have provided insight into Au(I) side effects and the optimal method of its administration [84–86]. AF, delivered orally, and Au(I) sodium thiomalate (GST), injected intramuscularly, were tested on patients. Both compounds cause gastrointestinal side effects, including diarrhea. They also cause aphthous ulcers, skin rash and pruritis, conjunctivitis and alopecia [84]. Although AF and GST exhibit comparable efficacy, AF causes less side effects [86]. Both are FDA approved (AF was approved in 1985) and have been used clinically to treat RA. Today the use of Au(I) compounds for this purpose is not common especially because their long term use, which is common due to their slow time for therapeutic effect, results in Au(I) itself triggering autoimmunity diseases [10].

### 3.3 Drug Repurposing of AF for Anticancer Application and an Evaluation of Its Mechanism of Action

At present, one of the mechanisms by which to expedite a promising compound to the drug market for a medical condition is to repurpose a known drug used for a different condition [87]. The benefits of this approach are numerous. Time, money, and human resources are decreased due to clinical trials already having been performed on the drug to examine its toxicity and to determine its safe dosage and optimal method of formulation and administration. AF is one such drug that is being repurposed for many different conditions [88,89]. Interest in AF for anticancer application stemmed from the finding that arthritic patients undergoing gold therapy had statistically lower incidences of cancer [10]. AF was found to be antiproliferative against many tumor cells in vitro [90]. In a study with 15 tumor models in mice, AF was found to be active against P388 leukemia and this was achieved via intraperitoneal administration [91]. A structure activity relationship study of 63 complexes of the general structural formula L–Au–X revealed that in vivo anticancer activity (especially against P388 leukemia) is generally optimized by ligation of Au(I) with a substituted phosphine (L) and thiosugar (X) [92].

**Figure 10.**
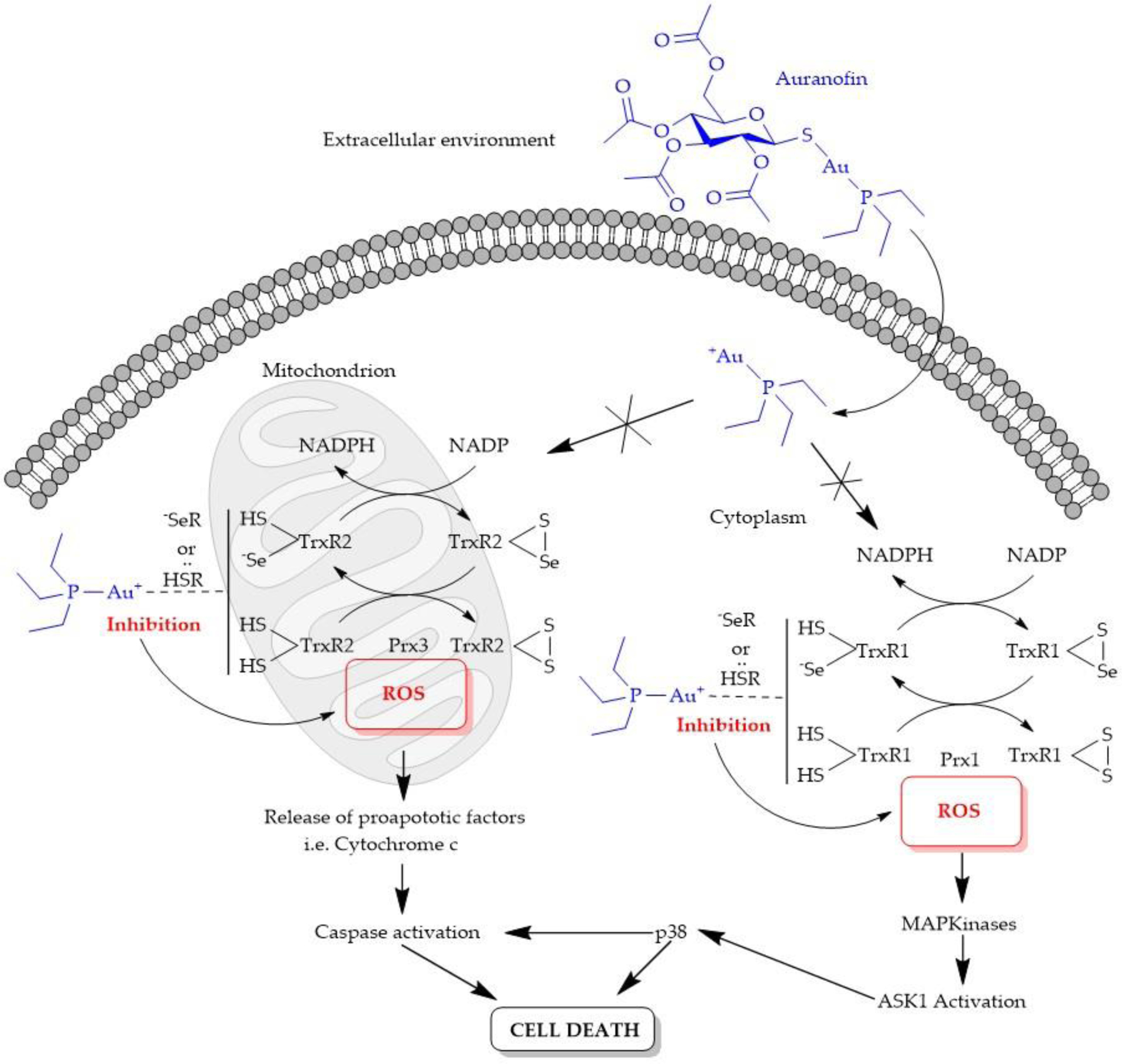
The proposed mechanism of cellular uptake and cytotoxicity of auranofin.

Like Cp_2_TiCl_2_, AF is classified a prodrug. Although Au(I) is able to bind to similar ligands as Pt(II) due to its Soft Lewis Acid nature, the proposed anticancer mechanism of action of AF and related Au(I) complexes is quite distinct from the Pt(II) drugs. DNA is not the primary target of these complexes. Their cytotoxicity is mediated by their ability to alter mitochondrial function and inhibit key enzymes by Au(I) binding strongly to functionally important thiol- or seleno-containing residues. Most of the thiol-containing enzymes, such as thioredoxin reductase (TrxR), glutathione reductase (GR), and cysteine protease, are overexpressed in cancer cells, thus providing potential anticancer targets for Au(I)-based therapy. Thiol groups are important in regulating mitochondrial membrane permeability. A study was performed on mitochondria isolated from rat livers [93]. In the presence of Ca^2+^ ions, Au(I) at submicromolar concentrations was observed to induce mitochondrial permeability transition [93]. In other words, the mitochondrial membrane becomes very permeable. This process appears to be triggered, in part, by Au(I) interfering with the thioredoxin redox system. The thioredoxin redox system comprises thioredoxin (Trx) and thioredoxin reductase (TrxR), which help to maintain redox balance within cells. Trx is found to reduce and activate many transcription factors involved in the regulation of cell growth and survival. Both Trx and TrxR are expressed as isoforms for cytosolic (TrxR1, Trx1) and mitochondrial (TrxR2, Trx2) localization [94]. These enzymes interact with one another in a tightly regulated redox process involving the formation of disulfide bonds in Trx and a sulfide and selenide bond in TrxR (Figure 10). As Au(I) binds tightly to thiol and selenol groups, it behaves as a potent inhibitor of Trx and TrxR. TrxR inhibition is particularly high (IC_50_ = 2.6 nM for purified enzyme) [95]. AF provokes an alteration of the redox state of the cell, leading to an elevated production of reactive oxygen species (ROS) beyond homeostatic control, which creates the conditions for apoptosis [94]. Apoptotic induction through a mitochondrion pathway is also observed with AF related complexes [96]. The inhibition of TrxR by these complexes can oxidize peroxiredoxin (Prx) enzymes. Peroxiredoxin 3 (Prx3) protects mitochondria from the ROS agent H_2_O_2_. This means that disturbance of Prxs can cause mitochondrial damage. High ROS levels disturbs the activity of cytosolic Prx1 and activates p38 MAPK, which can give a signal to the initiator caspase to trigger apoptotic events [94]. This trigger then leads to poly-ADP-ribose polymerase 1 degradation, DNA fragmentation, and finally, cell death. Due to the increased permeability of the mitochondrial membrane, the proapoptotic cytochrome c is released into the cytosol and contributes to signaling caspase-3 activation of apoptosis [95]. Despite DNA not being a direct target of Au(I), its replication can also be affected beyond fragmentation. AF can inhibit DNA polymerases essential for DNA replication if Au(I) binds to catalytically important sulfhydryl groups in these enzymes [89].

### 3.4 Auranofin Therapeutic Limitation due to Its Speciation in the Body

Various studies suggest that Au(I) from AF enters into cells via a sequential thiol exchange mechanism, where cell association of AF occurs from the shuttling of Au(I) between the sulfhydryl groups present on the cell resulting in the dissociation of the thioglucose moiety (Figure 9) [97,98]. The intracellular distribution of AF results from shuttling of membrane sulfhydryl bound gold-triethylphosphine to cytosolic sulfhydryl groups [98]. This would be a form of passive transport through the cell membrane. The triethylphosphine is believed to eventually dissociate in the process of Au(I) reaching its intracellular targets. Studies with a triple radioactively labeled auranofin, ^195^Au, ^35^S, and ^32^P, show different rates of clearance from the body. The t_1/2_ for urine excretion is Et_3_^32^P = O (8 h), ^35^S (16 h), and ^195^Au (20 days) [99]. These results ultimately suggest that AF is a prodrug.

The differences in the biological fate of the different components of AF could be due to additional factors involving AF speciation in the body and biomolecular interactions. Although AF is delivered orally, AF is actually not very water soluble. In the acidic conditions of the stomach, AF would rapidly degrade, leading to the loss of the thiosugar [100], but its relative insolubility may help to partially circumvent this issue. It is estimated that 25% of orally administered AF is absorbed from the gastrointestinal tract for distribution throughout the body [101,102]. AF cellular uptake competes with its serum speciation and this competition decreases its antiproliferative behavior [91],^99^,^100^]. In blood, AF rapidly interacts with the free cysteine (Cys34) residue of HSA, which results in the dissociation of the thioglucose, yielding a HSA{CysS–Au–PEt_3_}. It is not entirely clear whether HSA affinity for the [AuPEt_3_]^+^ moiety, hinders Au(I) interaction with and uptake by cell membranes. However, once the PEt_3_ ligand dissociates following HSA induced oxidation, forming Et_3_PO, the cytotoxicity of Au(I) is believed to be completely ablated. In the presence of the cyanide (CN^−^) ligand, Au(I) from AF quickly dissociates its phosphine and thiosugar ligands to form the highly stable [Au(CN)_2_]^−^ complex, which can bind to an anionic site of HSA. Though the cyanide ligand exists at very low amounts in the body, it is thought to play a major role in the wide distribution of [Au(CN)_2_]^−^ in the tissue of chrysotherapy patients [10] but likely serves to attenuate Au(I) cytotoxicity. Within cells, another biomolecular interaction can weaken the cytotoxic potential of AF. Glutathione (GSH), the cytosolic redox regulating small molecule, can strongly bind Au(I). GSH binding can induce the dissociation of the [Au-PEt_3_]^+^ moiety that manages to enter cells and can deter Au(I) interaction with cellular targets, in a manner analogous to its inhibition of Pt(II)-induced antiproliferation of cells [103].

### 3.5. Modifying the Phosphine and Thiosugar Ligands of Auranofin to Understand Their Mechanistic Contribution

The extreme lability of the Au(I) center limits the therapeutic potential of the prodrug AF and related Au(I) complexes. HSA binding of Au(I) as a consequence of the dissociation of the thiosugar ligand is a pivotal event for the attenuation of its cytotoxicity. A series of structure activity relationship studies have been performed to better understand the importance of the phosphine and thiosugar ligands to the bioactivity of Au(I). These studies provide insight into AF modifications that can make it a less ligand-exchange labile compound and subsequently less able to facilitate Au(I) binding by HSA in order to enhance its cytotoxic potency.

**Figure 11.**
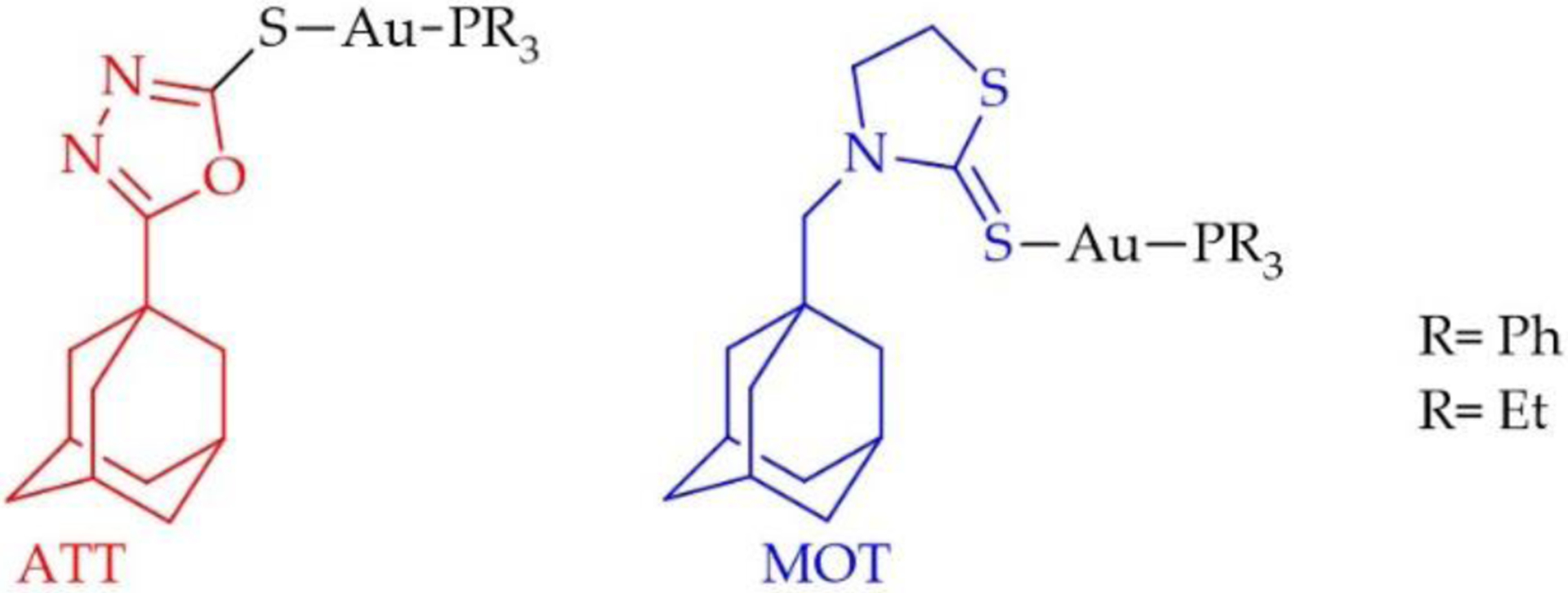
The Au(I) complexes with the sulfur coordinating groups 5-adamantyl-1,3-thiazolidine-2-thione (ATT) and 3-methyladamantane-1,3,4-oxadiazle-2-thione (MOT) and the phosphine ligands containing ethyl or phenyl substituents [104].

A recent study by Garcia et al. reveals very important ligand structural details that regulate the cytotoxicity of AF derivative complexes [104]. Four two coordinate, nearly linear Au(I) complexes were synthesized in which the phosphine group was either the PEt_3_ moiety or triphenylphosphine (PPh_3_) and the thiosugar was replaced with the sulfur coordinating groups 5-adamantyl-1,3thiazolidine-2-thione (ATT) and 3-methyladamantane-1,3,4-oxadiazle-2-thione (MOT) (Figure 11) [104]. Table 1 provides an overview of all the cell viability data collected for treatment of these compounds against the cancer cell lines B16-F10 (mouse metastatic melanoma), CT26-WT (murine colon), and 4T1 (mouse metastatic mammary adenocarcinoma) and the non-cancer cell line BHK21 (Baby Hamster Kidney) [104]. Quite apparent is that complete substitution of the thiosugar with a non-sulfur coordinating ligand as in AuPEt_3_Cl and AuPPh_3_Cl results in decreased cytotoxic potency by an average factor of 13 to 18 times. Garcia et al. rationalized that substitution of the thiosugar with the adamantyl containing ligands could increase the cytotoxicity because the adamantyl groups help the Au(I) complexes to bind at one of the two active sites of the TrxR enzyme, as shown by molecular docking, before direct Au(I) coordination occurs in the enzyme [104]. However, none of the four adamantyl-containing complexes demonstrated superior inhibitory effect toward TrxR than AF. Interestingly, AuPEt_3_MOT, in spite of displaying cytotoxicity comparable to AF, is the most promising derivative of this study because it exhibits a superior selectivity index (S.I.) for targeting the cancer cells (S.I. for AuPEt_3_MOT = 5.8 and S.I. for AF = 3.0). We calculated the selectivity index for each compound by dividing the IC_50_ value for BHK21 cells by the average IC_50_ value for the three cancer cell lines. The other three adamantyl-containing complexes exhibited inferior cytotoxicity and comparable S.I. to AF. There is no clear structure activity correlation with regards to the potential benefit of utilizing the adamantyl-containing ligands.

In the presence of 2 mg/mL of bovine serum albumin (BSA), which shares 80% sequence identity to HSA and can thus be viewed as nearly identical, almost all Au(I) complexes in Garcia et al.’s study exhibited anywhere from 10 to 100 times less cytotoxicity (as gauged by IC_50_ values) against the cancer and noncancer cell lines (Table 1) [104]. The decreased cytotoxicity effect was most pronounced for the noncancer cell line likely because, as previously mentioned, cancer cells catabolize albumin significantly more than normal cells [50]. Nonetheless, albumin debilitates the cytotoxicity of AF and related Au(I) complexes [50,105].

**Figure 12.**
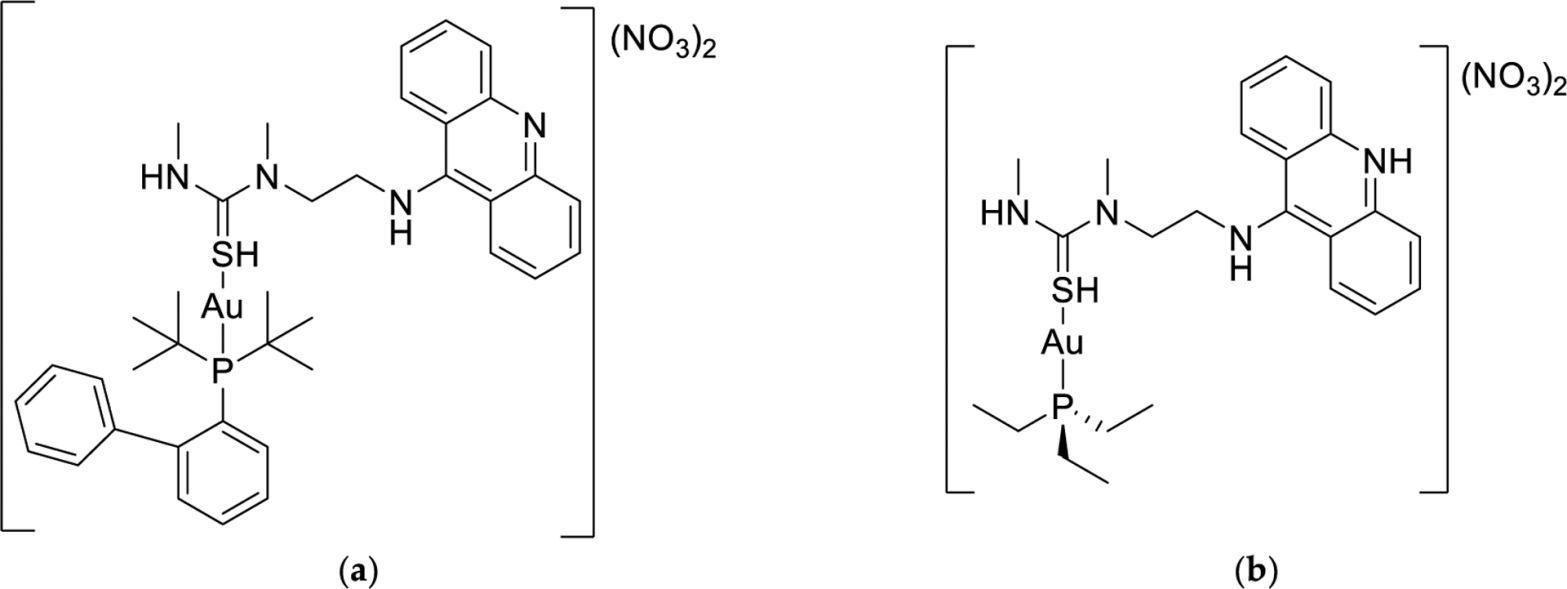
The chemical structures for a selection of AF derivatives containing the S-coordinating (1-[2-(acridin-9-ylamino)ethyl]-1,3-dimethylthiourea) (ACRAMTU) ligand and the 1,1′-biphenyl-2-yl]di-tert butylphosphine) (JohnPhos) (**a**) and triethylphosphine (**b**) [106].

**Figure 13.**
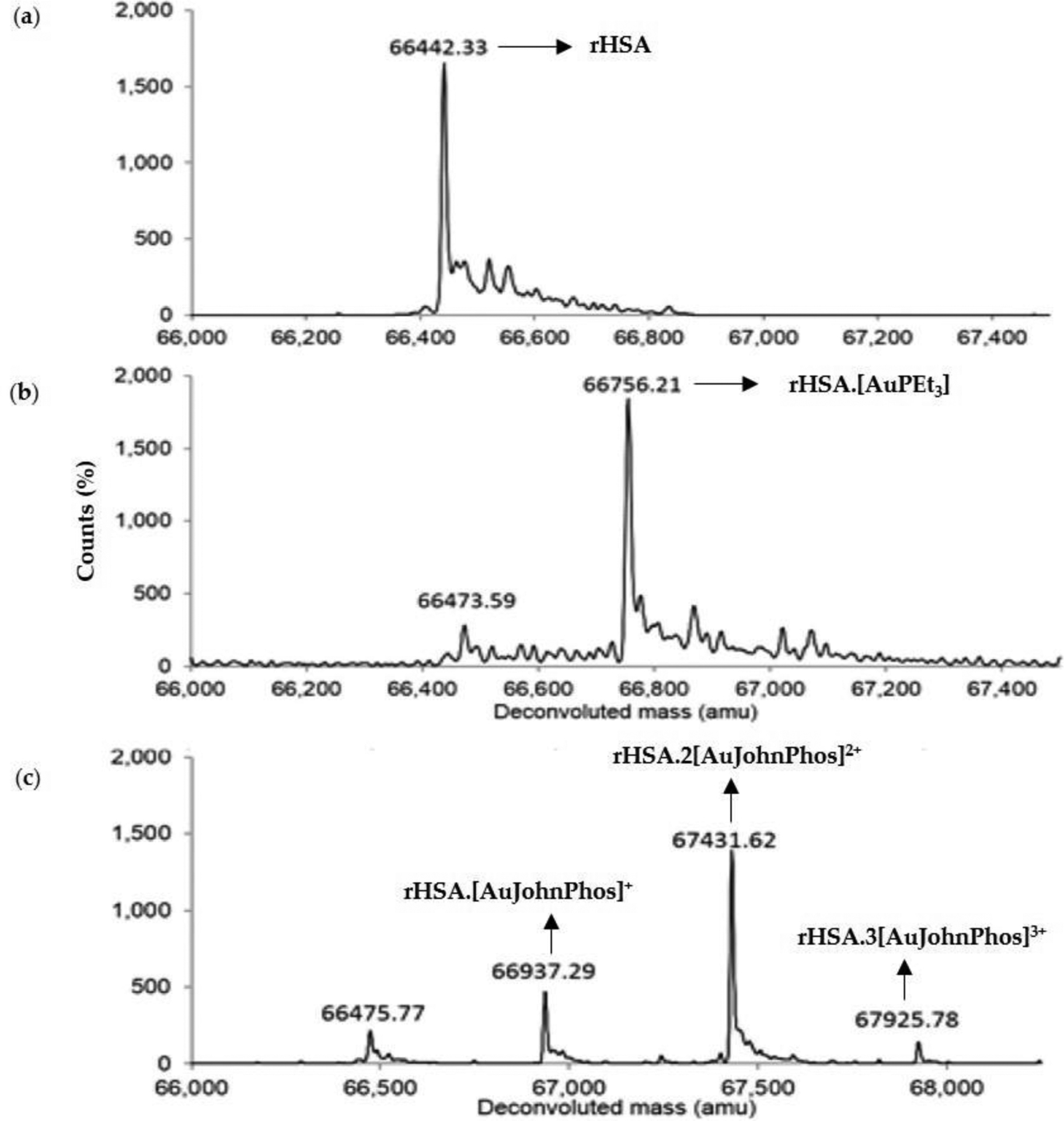
The ESI-TOF MS of free HSA (**a**) and HSA reacted with 1.33 equiv. of [AuPEt_3_]^+^ (**b**) and 1.75 equiv of [Au(JohnPhos)] (**c**) [107]. Adapted with permission from Dean, T.C.; Yang, M.; Liu, M.; Grayson, J.M.; DeMartino, A.W.; Day, C.S.; Lee, J.; Furdui, C.M.; Bierbach, U. Human Serum Albumin delivered [AuPEt_3_]^+^ is a potent inhibitor of T cell proliferation. *ACS Med. Chem. Lett*. **2017**, *8*, 572–576. Copyright (2017) American Chemical Society.

Substitution of the ethyl groups of the phospine ligand to the bulky aromatic groups in Garcia et al.’s study resulted in a decrease in cytotoxicity (Table 1) [104]. This decrease in cytotoxicity due to the incorporation of aromatic groups in the phosphine was also observed by Chaves et al. with the very similar Au(I) complexes containing both forms of the phosphine and 3-benzyl-1,3-thiazolidine-2-thione and 5-phenyl-1,3,4-oxadiazole-2-thione as the S-coordinating ligands [106]. A study by Dean et al. sheds some light as to why this substitution may be undesirable. In their work, they prepared Au(I) compounds with a series of thiourea molecules as the S-coordinating ligands and electron-rich diakyl-aryl phosphines (Figure 12) [107]. The hope was that the bulkier phosphine ligands would make them less labile to dissociation. In the presence of HSA, the S-coordinating ligands dissociate rapidly like the thiosugar of AF and the Au(I) phosphine moieties bind to HSA. Unexpectedly, the Au(I) diakyl-aryl phosphine moieties bind more extensively to HSA than [AuPEt_3_]^+^, which coordinates in a 1:1 protein:metal adduct, as monitored by electrospray ionization time of flight mass spectrometry (ESI-TOF MS) (Figure 13). The Au(I) complex with the 1,1′-biphenyl-2-yl-di-tert-butylphosphine and 5-phenyl-1,3,4-oxadiazole-2-thione as the S-coordinating ligands [106]. phosphine moieties (Figure 13) [107]. This study suggests that the addition of bulky, aromatic groups on the phosphine can result in greater interactions with albumin and subsequently, less cellular uptake of Au(I) and lower cytotoxicity.

**Figure 14.**
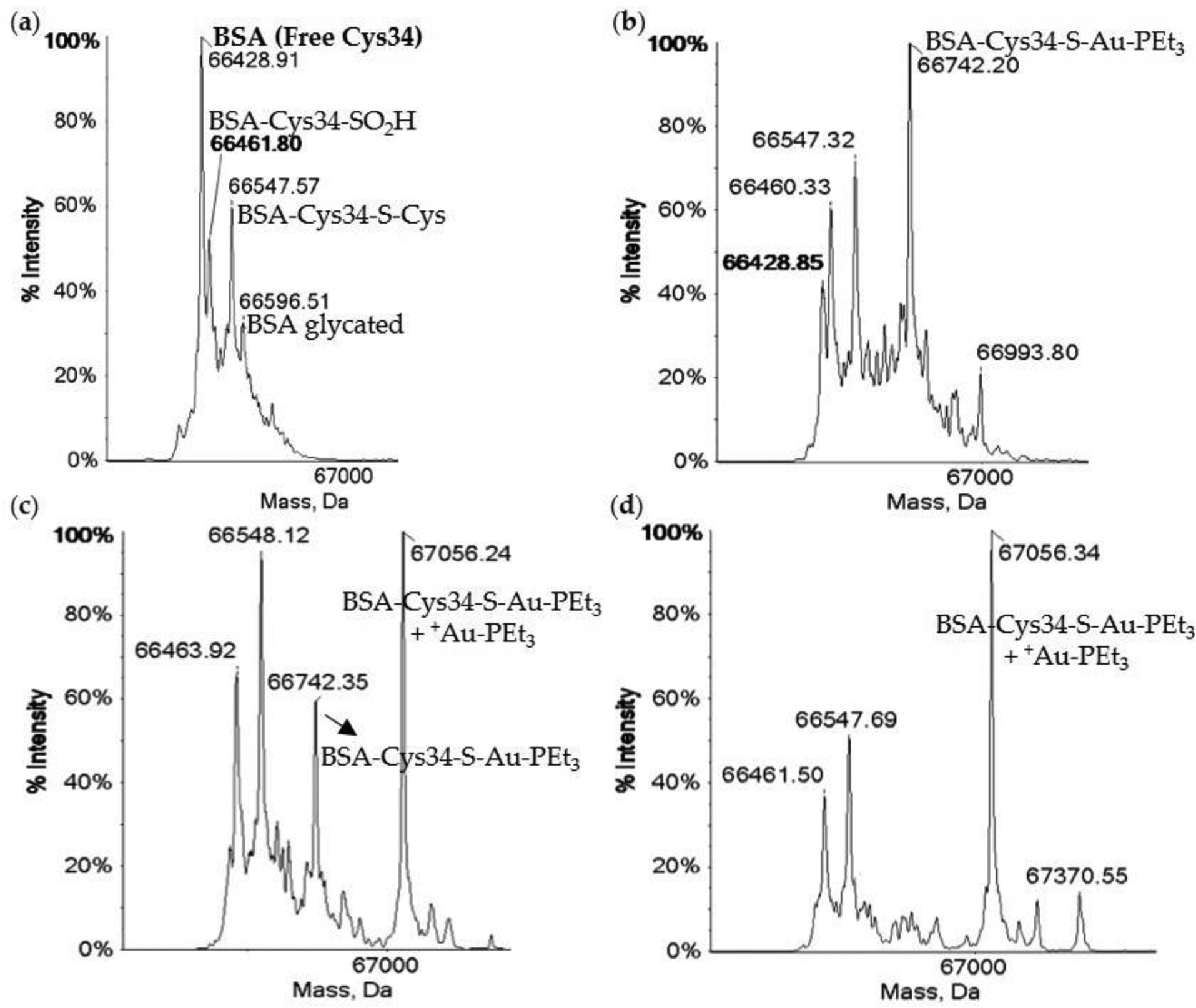
The ESI-MS of metal-free BSA (**a**) and BSA (100 μM) incubated with AuPEt_3_(CN) (**b**), AuPEt_3_(SCN) (**c**), AuPEt_3_(N_3_) (**d**) in a 3:1 metal to protein molar ratio after 1 h at pH 6.8 (20 mM NH_4_ Acetate) and 37 °C [108]. Note that metal-free BSA consists of several post translational modifications (PTMs) and only the Cys34 with no PTM is able to coordinate directly to Au(I). Also note that the incubation with AF produces an MS spectrum virtually identical to (**b**). Adapted with permission from Pratesi, A.; Cirri, D.; Ciofi, L.; Messori, L. Reactions of auranofin and its pseudohalide derivatives with serum albumin investigated through ESI-Q-TOF MS. *Inorg. Chem*. **2018**, *57*, 10507–10510. Copyright (2018) American Chemical Society.

Several studies demonstrate that the thiosugar of AF plays a more significant role than simply enabling sulfyl group exchange transport of the lipophilic [AuPEt_3_]^+^ moiety through the cellular membrane. Due to the high trans effect of the phosphine moiety of AF, the sulfur ligand, which is an excellent nucleophile, is more readily labile [19] than it would normally be. Modifying the nucleophilicity of the ligand trans to the phosphine can alter the ligand exchange reactivity of Au(I). Pratesi et al. conducted a study to examine how substitution of the thiosugar in AF with ligands of varying nucleophilicity would impact the interaction of Au(I) with BSA [108]. AuPEt_3_X complexes were prepared with the X ligands CN^−^, SCN^−^, and N_3_^−^, written in the order of decreasing nucleophilicity [108]. These complexes and AF were incubated with BSA in a 3:1 metal to protein molar ratio at pH 6.8 for up to 72 h. Their interaction with BSA was monitored by ESI-MS by measuring the intact protein mass and accounting for any mass differences that could be due to the formation of Au(I) adducts (Figure 14). It was observed that the AuPEt_3_X complexes reacted more extensively with BSA as the nucleophilicity of X decreased. The AuPEt_3_(CN) compound forms one adduct with BSA, the BSA{CysS34-Au-PEt_3_} species, as a consequence of the dissociation of the CN^−^ ligand. A similar result is observed for AF. The ESI-MS data for both complexes show that some amount of free BSA remains present, suggesting that perhaps a significant amount of the complexes may remain intact especially when considering that they were reacted in excess of the protein. The AuPEt_3_(SCN) compound forms both a mono[AuPEt_3_]^+^ and bis[AuPEt_3_]^+^ adduct (BSA{CysS34-AuPEt_3_^+^ [AuPEt_3_]^+^}) with BSA after 1 h. By 72 h (at equilibrium) only the mono [AuPEt_3_]^+^ adduct is observed, consuming all of the free BSA. The AuPEt_3_(N_3_) compound only forms the bis[AuPEt_3_]^+^ adduct with all of the available BSA.

**Figure 15.**
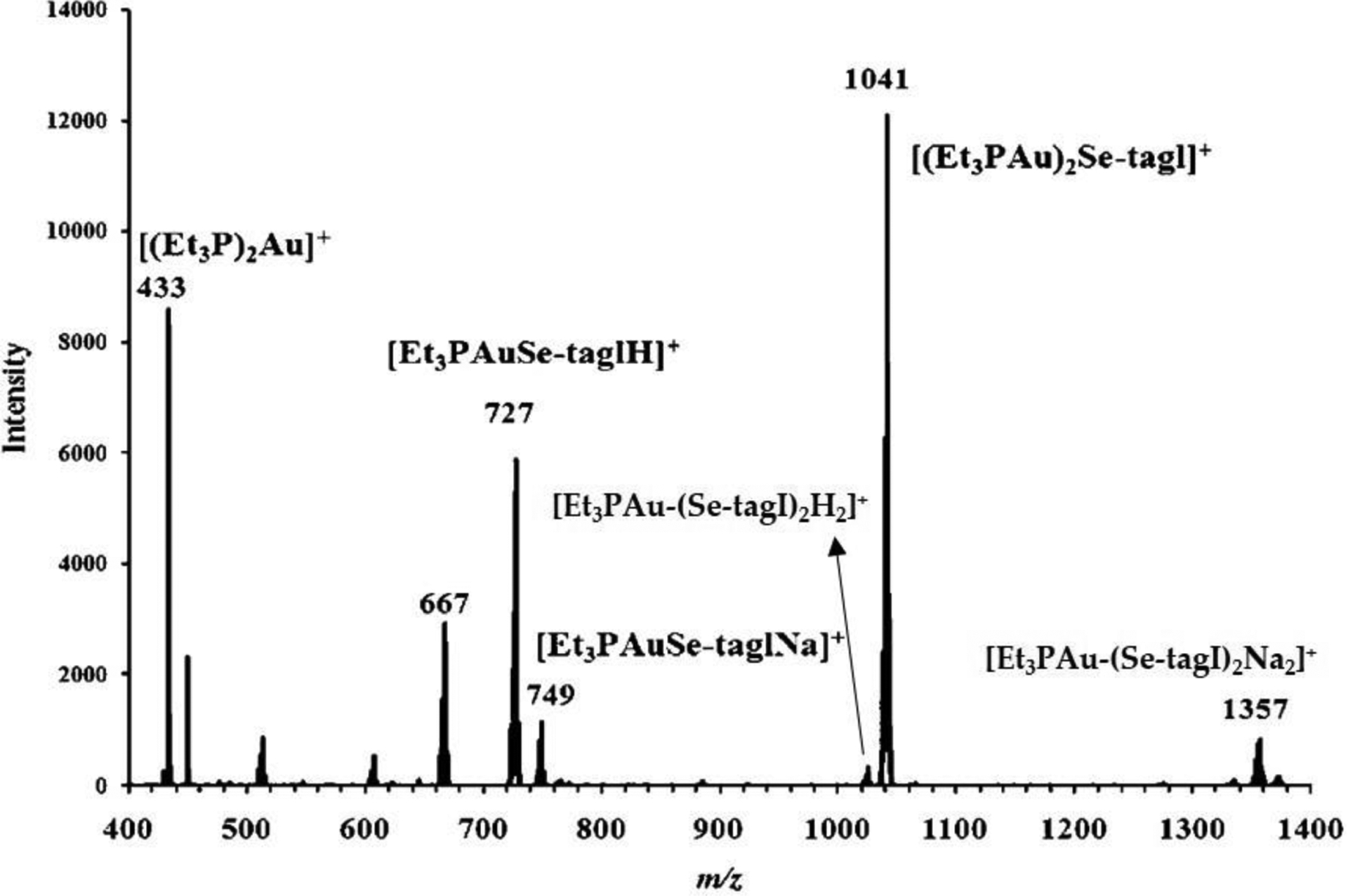
The ESI-MS spectra for the speciation of 10 mM SeAF (Et_3_PAuSe-tagl) in a 1:1 MeOH:H_2_O solution mixture [109]. Adapted with permission from Hill, D.T.; Isab, A.A.; Griswold, D.E.; DiMartino, M.J.; Matz, E.D.; Figueroa, A.L.; Wawro, J.E.; DeBrosse, C.; Reiff, W.M.; Elder, R.C.; Jones, B.; Webb, J.W.; Shaw, C.F. Seleno-auranofin (Et_3_PAuSe-tagl): synthesis, spectroscopic (EXAFS, ^197^Au Mossbauer, ^31^P, ^1^H, ^13^C, and ^77^Se NMR, ESI-MS) characterization, biological activity, and rapid serum albumin-induced triethylphosphine oxide generation. *Inorg. Chem*. **2010**, *49*, 7663–7675. Copyright (2010) American Chemical Society.

**Figure 16.**
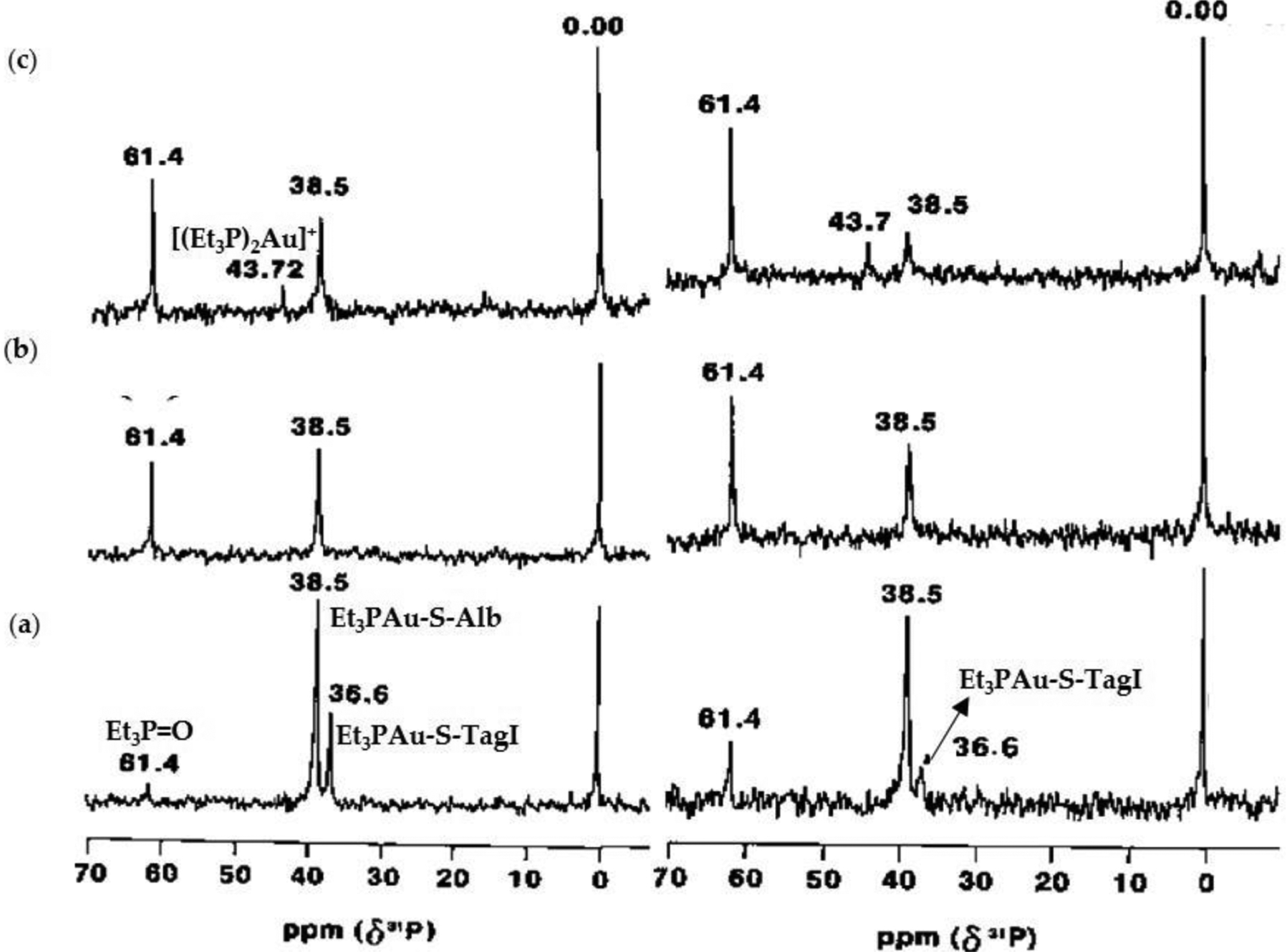
^31^P{^1^H} NMR spectra of 4.3 mM BSA (containing ~50% free Cys34) incubated with Et_3_PAuX (X = S-tagl, CN, Se-tagl) at 1:1 ratio with the free Cys34 and measured within 1 h (left) and after 24 h (right) at pH 7.9 in deuterated NH_4_HCO_3_ buffer; Et_3_PAuS-tagl (**a**), Et_3_PAuCN (**b**), and Et_3_PAuSe-tagl (**c**) [109]. Adapted with permission from Hill, D.T.; Isab, A.A.; Griswold, D.E.; DiMartino, M.J.; Matz, E.D.; Figueroa, A.L.; Wawro, J.E.; DeBrosse, C.; Reiff, W.M.; Elder, R.C.; Jones, B.; Webb, J.W.; Shaw, C.F. Seleno-auranofin (Et_3_PAuSe-tagl): synthesis, spectroscopic (EXAFS, ^197^Au Mossbauer, ^31^P, ^1^H, ^13^C, and ^77^Se NMR, ESI-MS) characterization, biological activity, and rapid serum albumin-induced triethylphosphine oxide generation. *Inorg. Chem*. **2010**, *49*, 7663–7675. Copyright (2010) American Chemical Society.

In 2010, Hill et al. substituted the sulfur of the thioligand of AF with selenium because of the stronger affinity that Se has for Au(I) than S [109]. This substitution had an effect opposite of what was intended. Seleno-auranofin (SeAF; Et_3_PAuSe-tagl) is extremely labile in aqueous solution, far more than AF, resulting in ligand scrambling and several species forming in solution as observed by ESI-MS (Figure 15) [109]. This lability is even more prevalent in the presence of albumin. ^31^P NMR reveals that albumin degrades SeAF more rapidly than AF (Figure 16) [109]. After one hour, the intact SeAF complex no longer exists (a small signal for one of its ligand scrambled products is observed) whereas the intact AF does. There is significant buildup of dissociated Et_3_PO in the SeAF reaction and little build up in the AF reaction, which suggests that the Se group accelerates the oxidation-induced release of the phosphine from Au(I) coordinated to the Cys34 site of BSA. By 24 h, the majority of the Et_3_P group from SeAF has transformed into Et_3_PO. In the case of AF, the majority of Et_3_P exists as the AuPEt_3_^+^ moiety bound to BSA although there is still some intact AF [109]. These time course results demonstrate that AF is far more labile exchange inert than originally thought.

**Figure 17.**
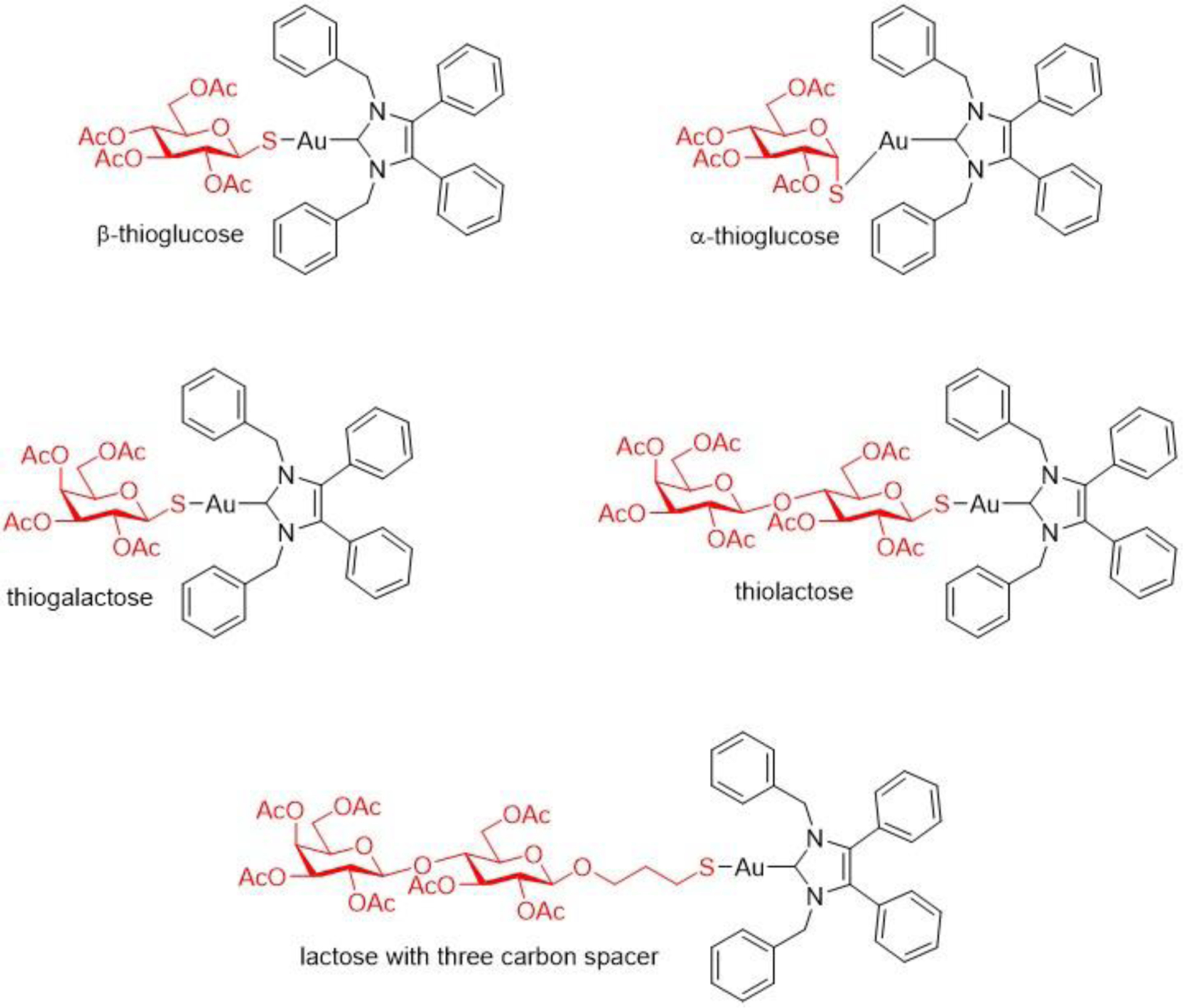
The structures of (1,3-dibenzyl-4,5-diphenyl-imidazon-2-ylidene)gold(I) complexes containing different sugars [110].

Recent efforts have tried to determine whether the thioglucose ligand may be able to facilitate a receptor mediated delivery of AF. The Warburg effect is the experimental observation that cancer cells prefer metabolism via glycolysis, thus, their rapid growth and proliferation usually involves glucose transporter (GLUT) overexpression and increased glucose uptake. This gives promise to the design of metallodrugs with glucose-like or glycomimetic moieties as semi-targeted anticancer treatments [110]. Walther et al. synthesized and studied the in vitro and in vivo anticancer properties of an AF analogue (NHC*-AuSR), in which a 1,3-dibenzyl-4,5-diphenyl-imidazon-2-ylidene ligand (NHC*) replaces AF’s triethylphosphine ligand (Figure 17). Nitrogen heterocyclic carbene (NHC) ligands have attracted much attention for the development of anticancer metallodrugs due to their high stability and ease of modification. The anticancer properties of the compound was compared to those of the chloro derivative (NHC*-AuCl) in order to determine if the thioglucose ligand enhances the compound’s cellular uptake through glucose transporters. Their antiproliferative potency against 60 cancer cell lines (the NCI-60 cancer cell line panel), their in vivo toxicity and tumor growth inhibition against a human renal cancer cell line (CAKi-1) tumor in a xenograft mouse model, and their TrxR inhibition were assessed. Also, computational molecular modelling was used to determine if the thioglucose analogue could be a glucose transporter ligand [111]. The results showed that both compounds exhibited very good cytotoxicity against a wide range of cancer cell lines, mostly at micromolar concentrations. In the tumor xenograft experiment, both compounds caused significant and almost identical tumor volume growth reductions while also being of low toxicity. Thirty five days after tumor transplantation, the tumor volume of the Au(I) compound treated mice was <50% that of the control group. Significant TrxR inhibition by both compounds (IC_50_ = 1.5 ± 0.2 μM for NHC-AuCl and 3.1 ± 0.4 μM for NHC*-AuSR) occurred. However, this inhibition was weaker than AF (IC50 = 90 nM) [111]. Like AF, the two Au(I) compounds enhanced oxidative stress resulting in apoptotic cell death. The molecular modeling results suggested that the thioglucose analogue could be a potential ligand for glucose transporter 1 [111].

To further study the role of the thioglucose ligand of the AF analogue, Dada et al. studied the replacement of this ligand with other sugars that have been conjugated to anticancer drugs in order to take advantage of the GLUT-mediated cellular uptake, which could potentially lead to a heightened activity [110]. In addition to AF’s β-thioglucose, the other sugar moieties they studied were α-thioglucose, galactose, lactose and also lactose with a three-carbon spacer between the sugar and the metal (Figure 17). All of the compounds were synthesized, characterized, and evaluated for their cytotoxicity against the NCI-60 cancer cell line panel. Generally, all of the synthesized compounds demonstrated good activity against the tested cancer cell lines at medium to low micromolar concentrations, while the α-thioglucose analogue exhibited the best activity overall at low micromolar to nanomolar concentrations. It should be noted that, in addition to the α-thioglucose analogue, the lactose analogue without the three-carbon spacer also exhibited better activity than the β-thioglucose analogue. It is remarkable how, in this case, simply changing the stereochemistry of the anomeric site of the original thioglucose ligand resulted in enhanced cytotoxicity against multiple cancer cell lines, even more than replacing the ligand for other sugar moieties that interact with GLUT. This could be considered a plausible modification to the β-thiosugar ligand of AF in order to potentially improve its cellular uptake via an alternative transport route and, consequently, its anticancer potency [110].

### 3.6 AF in Anticancer Clinical Trials

AF has advanced to clinical trials for its potential as a cancer chemotherapeutic [112]. The Mayo Clinic in Rochester, MI completed a Phase 1 trial to evaluate treatment of ten patients with asymptomatic ovarian cancer with cancer antigen 125 (CA-125) elevation [113]. The patients received 3 mg AF orally twice per day for 28 days. Some patients reported experiencing diarrhea, nausea, vomiting, fatigue, thrombocytopenia, and thromboembolism, none of which exceeded grade 2 of severity. Overall, AF was well-tolerated. Four patients manifested stable disease, one patient was removed from the study due to cancer progression, and one patient died. The median progression-free survival was 2.8 months. The clinic has move forward with a Phase 2 trial to determine whether the combination of AF and sirolimus can provide effective treatment of patients with ovarian cancer. The study is ongoing and involves 22 patients and the main objective is to evaluate the tumor response rate, progression-free survival, overall survival, and adverse events of participants. The Mayo Clinic in Arizona is performing a PhaseI/II trial on 47 patients to study the side effects and best dose of AF when given in combination with sirolimus to determine the effectiveness of this approach on lung cancer patients that have metastasized and cannot be treated with other methods or have experienced cancer recurrence. The University of Ulm School of Medicine is performing a proof of concept Phase 1 and 2 trial to evaluate the safety of the coordinated undermining of survival paths by 9 repurposed drugs (aprepitant, minocycline, disulfiram, celecoxib, sertraline, captopril, itraconasole, and AF) combined with metronomic temozolomide for recurrent glioblastoma. This trial will involve ten patients that undergo different cycles of the combination of temozolomide with the different repurposed drugs over the course of a year. The University of Kansas Medical Center performed a phase I/II clinical trial with five chronic lymphocytic leukemia (CLL) patients and one small lymphocytic lymphoma (SLL) patient. The patients were given 6 mg of AF daily for 28-day cycles, with a dose increase to 9 mg after the first cycle if no grade ≥2 toxicity was observed. The best response was stable disease. CLL cells were isolated from blood samples to evaluate the in vivo effect of AF. One day following the first dose administration of AF, there was an increase in the levels of ROS and similar increase in apoptosis. In addition, there were changes in genes regulating redox homeostasis. This is characteristic of the proposed mechanism of AF. However, by day 7, these changes reverted back to baseline. This suggests that there is an in vivo adaptive response to resist the effect of AF and consequently resulted in limited clinical activity of AF. No results were reported for the SLL patient [113]. The researchers plan to continue in vivo studies of AF on CLL patients at higher doses [114].

## 4. Fusing the Auranofin and Titanocene Moieties to Create a Bimetallic Complex with Heightened Cytotoxic Potency

**Figure 18.**
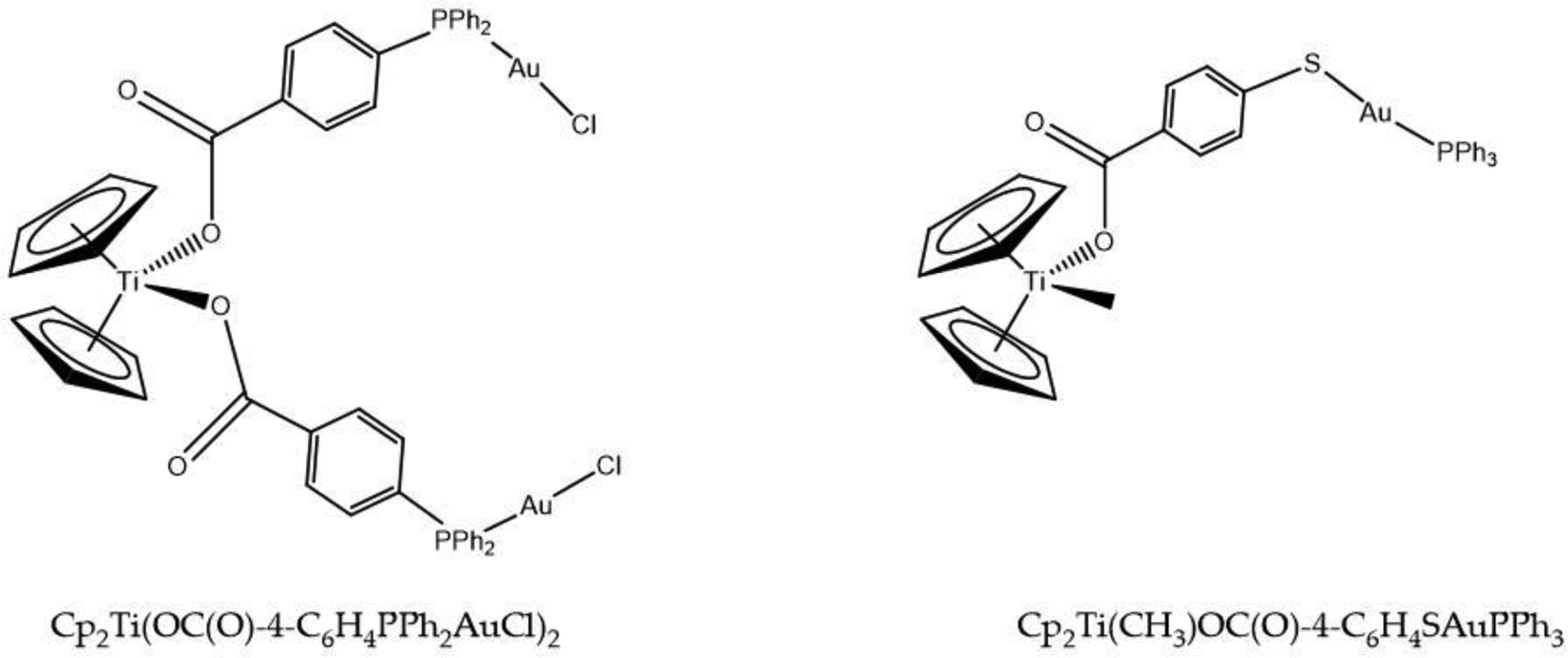
The chemical structures for Cp_2_Ti(OC(O)-4-C_6_H_4_PPh_2_AuCl)_2_ and Cp_2_Ti(CH_3_)OC(O)-4-C_6_H_4_SAuPPh_3_.

An intriguing approach to exploit the distinct cytotoxic mechanism of action of Ti(IV) and Au(I) is synergizing their activity in the form of heterometallic compounds [115–122]. These compounds build off of the titanocene structure. In the zero generation compounds, the gold binding moieties were conjugated to the Cp rings [118]. However, these compounds were not very stable in solution due to Cp ring dissociation. In the subsequent generations, the gold binding moieties were/are conjugated to groups that provide an oxygen ligation to the Ti(IV) and are far more stable [118]. Herein we will only focus on a select set of studies by Contel et al. centered on the fusion of the auranofin-like and titanocenyl moieties designed under the premise that two metals with different mechanism of actions will have a synergistic cytotoxic effect. In 2014, they prepared titanocene-gold complexes, one of which was the compound Cp_2_Ti(OC(O)-4-C_6_H_4_PPh_2_AuCl)_2_ (Figure 17) [116]. Treatment of several cancer cell lines with this heterometallic compound and the titanocenyl-free version demonstrated a profound synergistic decrease in the cell viabilities for the heterometallic compound (Table 2). This very significant synergism is something that would most definitely not have been achieved by mere co-treatment of the titanocenyl and Au(I) components of these compounds. From a surface perspective, this work demonstrates that two metals can be better than one in killing cancer cells. We believe that there is more at play. The choice of ligands and how they were designed to carefully coordinate both Au(I) and Ti(IV) possibly affords greater ligand exchange inertness to both metal ions and, as a positive consequence, decreased debilitating serum protein interaction. This is true even when the Cp rings dissociate. These results could explain the better activity exhibited by these compounds versus the zero generation compounds and the enhanced synergistic effect. A 2015 follow-up study was performed with a heterometallic compound Cp_2_Ti(CH_3_)OC(O)-4-C_6_H_4_SAuPPh_3_ (Figure 18), which features Au(I) in a ligation more analogous to AF [117]. This compound is very potently cytotoxic against the cancer cell lines examined (IC_50_ ~ mid nM to very low μM). Cellular uptake of the compound in Caki-1 cells (human renal cancer) demonstrated that the compound is quite robust as it appears to enter intact, with co-localization of both metals and a dose-dependent increase in their levels. This apoptotic compound features the signature activity of AF by inhibiting thioredoxin reductase and of Cp_2_Ti^2+^ in being unable to induce a direct DNA strand break [117]. In vivo studies also showed an important decrease of tumor size by 67% compared to the control, supporting the in vitro studies in which this compound is able to block the growth of renal cancer [114]. More recent studies in 2019 showed that Cp_2_Ti-OC(O)-4-C_6_H_4_SAuPPh_3_ (Figure 18) and an ethyl derivative Cp_2_Ti-OC(O)-4-C_6_H_4_SAuPEt_3_, can attack more than one characteristic of cancer such as migration, invasion and angiogenesis while being more selective that monometallic counterparts [121]. These heterometallic Au(I) and Ti(IV) hold great potential for clinical screening.

## 5. Conclusions and Future Directions

Empirically driven research has led to many breakthroughs in the medical field. It brought the serendipitous finding of the anticancer properties of cisplatin, which gave birth to a scientific Renaissance to explore other metals in medicine that led to the discoveries of Cp_2_TiCl_2_ and AF. Like cisplatin, Cp_2_TiCl_2_ and AF are classified as prodrugs. The distinct metal centers of these compounds confer different coordination chemistries and interactions with biomolecules, which subsequently results in different effects on cells. However, by dissecting the biological speciation of these compounds and their mechanism of action within cells and in vivo, much insight has been gained about the limitations posed to their therapeutic potential and about how the ligands can be fine-tuned to play a more active role to increase potency and specificity for cancer cells. It has become more apparent that decreasing the ligand exchange lability of the titanocenyl moiety and the thio and phosphine ligands of AF is essential as is altering structural features of these ligands to minimize serum protein interactions that hinder their cellular uptake and/or alter their speciation by producing noncytotoxic species. In the case of the titanocene family of compounds, the titanocenyl moiety may in fact be very important to damaging DNA and leading to cell death and so minimizing the synergistic destabilization of these compounds by STf and citrate is necessary. For the AF family of compounds, preserving the core ligation is needed to prevent HSA induced dissociation of the compound. Functionalizing the ligands of both compounds with biological vectors or inorganic nanoparticles can aid in facilitating alternative cell uptake routes that can result in higher cellular levels of intact compounds. Deviating from the core ligation of the AF and Cp_2_Ti^2+^ moieties can dramatically alter the cellular effect of Au(I) and Ti(IV) by not only improving the ligand-dissociation stability and biomolecular interactions of the metal ion [10,77,123–125] but also potentially changing the intracellular targets of the metals [17,23,125–127].

## Figures and Tables

**Table 1.** Antiproliferative activities of Au(I) complexes against cancer cell lines and inhibition at 5 μM of TrxR activity [104].

Compounds	Cancer Cells IC_50_ (μM ± SD)			Non-Cancer Cell BHK21	TrxR Inhibition (% ± SD)
	B16-F10 Melanoma	CT26-WT Colon	4T1 Breast		
AuPPh_3_ATT	5.7 ± 0.5	5.7 ± 0.9	6.6 ± 0.5	18.5 ± 2.9	40.7 ± 1.5
AuPEt_3_ATT	1.2 ± 0.2	1.8 ± 0.8	1.6 ± 0.5	5.5 ± 0.1	51.6 ± 1.6
AuPPh_3_MOT	1.8 ± 0.5	1.8 ± 0.3	3.0 ± 1.8	6.9 ± 0.8	57.3 ± 0.7
AuPEt_3_MOT	1.0 ± 0.1	0.9 ± 0.1	1.1 ± 0.2	5.8 ± 0.1	60.2 ± 0.7
AuPPh_3_Cl	6.6 ± 0.1	12.1 ± 2.8	10.3 ± 2.3	23.0 ± 0.3	-
AuPEt_3_Cl	2.3 ± 0.5	9.0 ± 0.7	9.5 ± 1.1	22.5 ± 0.2	-
AF	0.5 ± 0.4	0.5 ± 0.4	0.6 ± 0.2	1.6 ± 0.5	59.7 ± 1.5

**Table 2.** Antiproliferative activities (μM ± SD) of Ti(IV)–Au(I) complexes against cancer cell lines.

Compounds	A498 Kidney	UO31 Kidney	Caki-1 Kidney	HEK-293T Kidney	PC3 Prostate	DU145 Prostate
Cp_2_Ti-(OC(O)-4-C_6_H_4_PPh_2_AuCl)_2_	6.9 ± 2.2	0.3 ± 0.06	1.0 ± 0.29	20.1 ± 1.6	37.7 ± 7.1	6.6 ± 1.8
HOC(O)-4-C_6_H_4_PPh_2_AuCl	21 ± 2.5	1.2 ± 0.8	19.2 ± 2.9	31 ± 0.9	78 ± 18.1	39 ± 5.7
Cp_2_Ti-OC(O)-4-C_6_H_4_SAuPPh_3_	ND	ND	0.12 ± 0.003	0.49 ± 0.008	ND	ND
C_6_H_4_SAuPPh_3_	ND	ND	2.76 ± 0.35	1.11 ± 0.65	ND	ND
Cisplatin	37.2 ± 4.6	8.9 ± 2.7	29 ± 4.1	3.2 ± 0.13	14 ± 2.3	12.1 ± 3.9
Cp_2_TiCl_2_	>200	>200	>200	>200	>200	>200
Titanocene Y	29.6 ± 2.8	>200	29.4 ± 4.2	>200	58.1 ± 11.2	55.2 ± 7.9
